# Assessing Music Perception in Young Children: Evidence for and Psychometric Features of the M-Factor

**DOI:** 10.3389/fnins.2017.00018

**Published:** 2017-01-24

**Authors:** Caio G. Barros, Walter Swardfager, Sylvain Moreno, Graziela Bortz, Beatriz Ilari, Andrea P. Jackowski, George Ploubidis, Todd D. Little, Alexandra Lamont, Hugo Cogo-Moreira

**Affiliations:** ^1^Music Department, State University of São Paulo (UNESP)São Paulo, Brazil; ^2^Department of Pharmacology and Toxicology, University of TorontoToronto, ON, Canada; ^3^Hurvitz Brain Sciences Program, Sunnybrook Research InstituteToronto, ON, Canada; ^4^School of Engineering, Simon Fraser UniversitySurrey, BC, Canada; ^5^Thornton School of Music (Program of Music Teaching and Learning), University of Southern CaliforniaLos Angeles, CA, USA; ^6^Department of Psychiatry and Medical Psychology, Federal University of Sao PauloSão Paulo, Brazil; ^7^Department of Social Sciences, Center of Longitudinal Studies, University College of London (Institute of Education)London, UK; ^8^Institute for Measurement, Methodology, Analysis, and Policy, Texas Tech UniversityLubbock, TX, USA; ^9^Faculty of Natural Sciences and School of Psychology, Keele UniversityKeele, UK

**Keywords:** assessment, psychometrics, hearing, children, music perception, bifactor model

## Abstract

Given the relationship between language acquisition and music processing, musical perception (MP) skills have been proposed as a tool for early diagnosis of speech and language difficulties; therefore, a psychometric instrument is needed to assess music perception in children under 10 years of age, a crucial period in neurodevelopment. We created a set of 80 musical stimuli encompassing seven domains of music perception to inform perception of tonal, atonal, and modal stimuli, in a random sample of 1006 children, 6–13 years of age, equally distributed from first to fifth grades, from 14 schools (38% private schools) in So Paulo State. The underlying model was tested using confirmatory factor analysis. A model encompassing seven orthogonal specific domains (contour, loudness, scale, timbre, duration, pitch, and meter) and one general music perception factor, the “m-factor,” showed excellent fit indices. The m-factor, previously hypothesized in the literature but never formally tested, explains 93% of the reliable variance in measurement, while only 3.9% of the reliable variance could be attributed to the multidimensionality caused by the specific domains. The 80 items showed no differential item functioning based on sex, age, or enrolment in public vs. private school, demonstrating the important psychometric feature of invariance. Like Charles Spearman's g-factor of intelligence, the m-factor is robust and reliable. It provides a convenient measure of auditory stimulus apprehension that does not rely on verbal information, offering a new opportunity to probe biological and psychological relationships with music perception phenomena and the etiologies of speech and language disorders.

## Introduction

Accurate measurement of music perception (MP) domains, such as pitch, rhythm, and meter is central to understanding the brain processes that underlie musical behavior. Such behaviors may have emerged early in primate evolution, since studies of pitch perception (e.g., the pitch strength of a harmonic tone dominated by resolved harmonics) suggest that marmosets and humans share a common pitch perception mechanism (Song et al., 2016). The neural coding of pitch, a primary auditory sensation, is of practical importance, for instance in the design of neurobionic therapies for hearing loss (Tramo et al., 2005). At present, cochlear implant users, and to some extent hearing aid users, struggle with complex auditory perceptual tasks, particularly those requiring perception of pitch (Looi et al., 2015) and melodic contour (See et al., 2013). In some studies, even short periods of training can strongly influence the functional organization of the developing brain, enhancing pitch discrimination abilities in speech (Santos et al., 2007; Moreno et al., 2009). Accordingly, the perception of pitch contour in spoken language differs between musicians and those without musical training (Schön et al., 2004) and perception of musical pitch and temporal processing account for 34.5% of the variance on speech prosody test performance (Morrill et al., 2015).

Compared to age-matched controls, children with specific language impairment (SLI) exhibit deficits in rhythmic cues in speech and music, as evaluated by beat detection tasks (Cumming et al., 2015), and dyslexic children may have difficulties discriminating strong pitch changes that are easily discriminated by normal readers (Besson et al., 2007). Moreover, production of complex syntax and reorganization of grammatical information have been related to rhythm perception, indicating that grammar and rhythm share some degree of cognitive resources (Gordon et al., 2015). The relation between language acquisition and music processing has led (Sallat and Jentschke, 2015) to propose that musical material could be used for early diagnosis of SLI.

As processing of prosodic information involves similar skills to those required in MP (Sallat and Jentschke, 2015), and emerging evidence supports a common cerebral network involved in both lexical/phonological and melodic processing (Schön et al., 2010), MP may offer a useful universal non-verbal marker for language acquisition. Therefore, as opposed to language tests centered on verbal skills, MP may inform a more general understanding of speech and language disorders. An accurate standardized assessment of MP skills would offer new opportunities to probe non-verbal auditory skills as pitch, meter, and melodic contour; however, few tests, batteries, or scales have been developed for use in children under 10 years of age, a crucial neurodevelopmental period.

Research conducted predominantly in the Western world suggests that music perceptual abilities develop considerably over the course of childhood. Humans are known to enter the world with some remarkable abilities to perceive pitch and rhythm. Infants discriminate pitch changes in a short familiar melody (Fancourt et al., 2013), and they can discriminate between consonance and dissonance (Trainor and Heinmiller, 1998; Zentner and Kagan, 1998). Preschoolers can also make discriminations based on pitch changes (Fancourt et al., 2013), and are sensitive to musical consonance and dissonance in both behavioral (Trainor and Corrigall, 2010) and brain studies (Koelsch et al., 2003). While frequency discrimination and pitch change detection become adult-like at about 6–7 years of age, sensitivity to pitch direction and harmonic perception reach adult levels only at around 10 or 11 years of age (Trainor and Corrigall, 2010; Fancourt et al., 2013). In terms of rhythmic perception, infants can readily discriminate between short contrasting rhythmic patterns (Trehub and Trainor, 1998), as well as inferring metrical structures in a listening context (Hannon and Trehub, 2005). Children have been shown to attend simultaneously to pulse and rhythm between 5 and 7 (Paananen, 2006); more recent data have shown that 5-year-olds were significantly better able to detect beat misalignments in music in simple than in complex meter (Einarson and Trainor, 2016).

Regarding the evaluation of music skills among young populations, the first MP test was developed by Wing (1948) to assess acuity of musical hearing and sensitivity to performance beginning at 8 years of age. Gordon (1986) described three batteries: the Musical Aptitude Profile (for fourth grade students, consisting of seven subtests including both tonal and rhythm dimensions), the Primary Measure of Music Audiation (designed for students below third grade), and the Intermediate Measures of Music Audiation (for fourth grade students). The intermediate measures are similar to the primary measures but include more difficult items. Recently, Peretz et al. (2013) introduced the Montreal Battery of Evaluation of Musical Abilities (MBEMA), comprised of tests of memory, scale, contour, interval, and rhythm, administered to 245 children in Montreal.

In general adult populations, the Profile of Music Perception Skills (PROMS), proposed by Law and Zentner (2012), assesses musical ability under two higher order factors (i.e., sequential and sensory music processing). The Clinical Assessment of Music Perception (CAMP) was developed by Kang et al. (2009) to evaluate MP in adults with cochlear implants. The Montreal Battery Evaluation of Amusia (MBEA), proposed by Peretz et al. (2003), has emerged as the “gold standard” to assess Amusia (Wilcox et al., 2015), being the most widely used tool for the evaluation of musical disorders in adults (Stewart et al., 2006). This latter battery has proven informative in several different sub-disciplines, for example, to specify musical difficulties in subtypes of amusia (Sloboda et al., 2005; Pfeifer and Hamann, 2015; Wilcox et al., 2015), and to demonstrate relationships between music and speech perception (Hausen et al., 2013) and auditory sensory processing (Korzyukov et al., 2012).

Existing music perception batteries for both children and adults are generally composed of items that require accurate discrimination of pitch, contour, scale and meter. Law and Zentner (2012) hypothesized the existence of a general factor for MP encompassing all of these domains, but that assertion has not been formally tested. Here we describe a new set of 80 musical stimulus items (composed by CGB) that assesses seven domains (contour, pitch, scale, duration, dynamics, meter, and timbre), in a manner suitable to formally test whether all of the items will inform a measurable general MP factor that reflects the variability in responses common among all items.

Commonly, specific domains of music perception are measured using subscales derived from various instruments, and their correlations are assessed with neurobiological features or with linguistic skills; however, no evidence has been provided to support the reliability or viability of those subscales. Moreover, robust psychometric studies validating available MP scales, batteries and tools, and describing the latent structure of MP, remain sparse. Through more sophisticated aspects of item response theory, we can evaluate how the variance common to only the items designed to measure the same specific factor (e.g., a “pitch” subscale), can explain variance in the item responses that is not better accounted for by the general factor. Specifically, the bifactor model has emerged as a tool to evaluate hypothetical models comprised of two parts, a more general factor (i.e., music perception) and more specific factors (e.g., subscales related to pitch, contour, scale, etc.).

**Figure 1:**
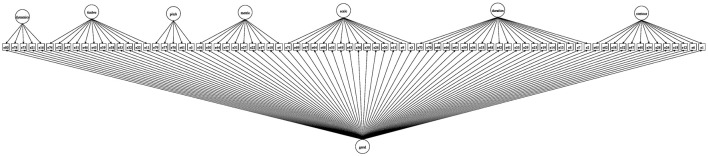
**Diagram representing the bifactor model's structure underlying the 80 items of the music perception test**.

The first aim of this study is to confirm that the proposed battery conforms to the hypothesized bifactor model, unifying the responses to each item to estimate a general MP factor, here called the “m-factor,” along with the seven specific factors proposed. The hypothesized conceptual bifactor model is shown in Figure 1. The bifactor model concomitantly evaluates the viability and reliability of variances attributable to both the general and specific factors (see Methods).

The secondary aim of this study is to show that the proposed battery performs similarly across the population regardless of differences in common demographics unrelated to MP. Although this psychometric feature is of fundamental importance, especially for cross-cultural comparisons and comparisons between subgroups (e.g., between boys and girls), to our knowledge invariance has not been evaluated for any of the available MP batteries.

## Methods

### MP battery

Eighty pairs of stimuli (items E1–E80 described musically in Image 1 in Supplementary Material) were composed based on the two alternative forced choice paradigm, as in the majority of previously presented batteries, where the child decides if a pair or stimuli are equal or different. The 80 pairs were designed to evaluate seven MP subdomains: contour (13 items), timbre (12 items), meter (10 items), pitch (5 items), scale (15 items), duration (20 items), and loudness (5 items):

- **Contour**: we used continuous and discrete sounds, many types of pitch structures (e.g., major and minor scales for E1 and E6, respectively, and a 12-tone row for E12), different places in the instrument range, and so on. Other compositional strategies were considered in domains, such as: the same note in a different octave, a different note in the same diatonic scale, percussion with indefinite pitch (woodblocks), a different note in an atonal pitch set, and a synthesized saw-tooth waveform in continuous lines (glissando).
- **Pitch**: in our model differentiation between high and low notes should be independent of determination of pitch. To measure this, it is important to keep sounds with definite and indefinite pitch in the same subtest. We used a sampled piano tone, a saw-tooth waveform, and filtered white noise.
- **Scale**: we used three paradigms based on historical musical systems in Western music: modal, tonal, and atonal (e.g., Ionian/major on E3, Lydian E9, Dorian on E13, Octatonic on E67, diatonic vs. chromatic/atonal difference on E71). “Atonal” is actually not a systematized musical code, but uses musical parameters that contradict the common patterns of the previous two. We did not alter contour/shape for any of the items, in order to isolate the aspect of scale.
- **Duration**: In this domain, we changed note durations but maintained the meter, where applicable. Changes in tempo, and simple comparisons of two sounds were also included (e.g., E42 and E53, respectively). We used percussion with undefined pitch (snare drum and woodblock), sampled piano, flute, a synthesized saw-tooth waveform, and white noise.
- **Dynamics (loudness)**: synthesized saw-tooth waves and white noise were used. We used both prolonged and “percussion type” sounds. The sounds differed only in wave amplitude.
- **Meter**: We used sampled cowbell, bass drum, guitar (a synthetic one and one recorded to include the effect of rasgueado in muted strings in E17), piano, woodblocks, violin and flute. Most of the items contain binary or ternary metrics and two contain meters with 5 (E50) and 7 beats (E56). We used a wide variety of timbres and instruments with defined and undefined pitches in order to measure perception of meter in different ways.
- **Timbre**: The changes in timbre in our items were mostly created with electronic effects. We used high and low-pass filters and reverberation. This procedure differs from that used in the construction of the PROMS (Law and Zentner (2012), in which changes in timbre were created by changing instrument groups playing a chord. In the present battery, only one item (E39) had two instruments (cymbal and tom-tom) in the first stimulus, with the tom-tom removed in the paired stimulus. Some items with synthesized sounds were included with “prolonged” vs. “percussive”' sound types (E38). Again, a wide variety of sampled instruments and synthesized sounds were used, including piano, cymbals, tom-tom drums, filtered white noise, sinusoid waves, oboe and timpani. A comprehensive measurement of timbre is challenging and would require extensive separate study. For instance, Schaeffer (1966) built a highly complex theoretical framework for the study of “sound objects” that greatly surpasses what is possible in our battery.

An important difference between the proposed battery and previous batteries is the inclusion of items beyond the paradigm of tonality. Most previous batteries, such as the MBEMA (Peretz et al., 2013) and The Primary Measure of Music Audiation (Gordon, 1986) consist exclusively of stimuli built around tonality; however, there is little *a priori* reason to restrict items to a single musical code. Moreover, increased variety will permit exploration of how each item and its respective parameters behave psychometrically within a multidimensional model. Although atonal stimuli are included in the PROMS (Law and Zentner (2012), these are restricted to the set of melody subtests, and are restricted to free chromaticism (i.e., using the 12 tones of the Western chromatic scale with no particular underlying structure), a likely consequence of the authors' choice of “basic,” “abstract,” and “stylistically neutral” stimuli. As seen in the description of the items in each domain above, we deliberately chose musical paradigms based on historical systems of Western music: tonal, atonal and modal, as well as sounds without defined pitch (i.e., percussion and electronic noise) to circumvent such restrictions in a systematic way. Moreover, while the MBEMA uses exclusively synthesized piano sounds, and the Primary Measure of Music Audiation uses sinusoidal waves, we adopted a wide range of timbres (i.e., different musical instruments, electronic sounds, filtered white noise, etc.) in order to be less restrictive.

Finally, by having a systematic set of musical paradigms as the basis of stimulus composition, we take into account the particularity of each domain in the manipulation of musical parameters. For example, while rhythmic changes in all three of the previously cited batteries have been associated with strong and weak beats in musical measures, we manipulated sound durations, and in the domain of meter, we changed types of meters (from binary to ternary and vice versa) including “unusual” meters, such as 5 and 7 beats. We also used different types of scales and continuous sounds (*glissandi*) in the domain of contour, to afford increased opportunity to assess musical discrimination. Details of the musical modes of the stimuli and their paradigms (i.e., equal or different) are shown in **Table 2** (left side).

The proposed battery can be administered within 30 min, as suggested by teachers in a pilot study to avoid interference due to attention. Among adults, active listening tasks of even a few hours can lead to reorganization of certain cortical representations (Pantev et al., 1999), and although evidence is lacking for children, restricting the number of items may be advisable to avoid practice effects.

The following software were used: Rosegarden for midi tracks, Linux Sampler for sampling, Piano and instruments gig: Maestro Concert Grand V2 (piano), Philharmonia Orchestra (percussion, clarinet and guitar), Vienna Symphonic Orchestra Pro (Oboe, flute, violin), Pure Data for sound synthesis and filtering (Puckette, 2007), Lilypond for score engraving (Nienhuys and Nieuwenhuizen, 2003), and Snd to create wave and spectral graphs.

### Evaluator training

Fourteen evaluators (one per school) undertook training to ensure that equivalent instructions were given. Providing feedback to participants in the form of demonstrative facial signs or oral positive/negative cues was discouraged. To counter loss of attention or demotivation, the motivational phrases “you are almost finished,” “do not give up” and “it is almost complete” were used. Phrases, such as “you are doing well” were discouraged, as they could be perceived as feedback in relation to the answers given and thus interfere with performance.

### Application of the battery

This research was approved by the Ethical Committee from the Federal University of Sao Paulo (CAEE: 00751812.3.0000.5505). Written informed consent was given by parents or children's legal guardians.

All children were tested alone in a quiet setting at school using Philips headphones (model SHL 9560). Prior to beginning the test, a standardized set of instructions was read by the evaluator. Six pre-test stimuli were played to evaluate whether the child fully understood the instructions:

“You, [child's name], will hear two sound sequences separated by a short silence. You should decide if these sound sequences are the same or different. Then, press the *different* button if the two sequences are at all different, no matter how small. Press the *equal* button if you believe that the two sequences of sounds are exactly the same.”

During the six pre-test stimuli, the evaluator was permitted to help the child, explaining why and when the stimulus pairs were the same or different. The six pre-test stimuli and instructions were repeated until the child understood; for these six pre-tests, the program gives feedback for the evaluator and child, informing them if the response was correct or not.

The 80 items from the battery were then played, and responses were recorded by the software as 1 if the question was correctly answered and 0 if answered incorrectly. The child and evaluator did not receive any feedback about how many items were marked correctly or incorrectly. There was no limit to the amount of time allowed for the child to press the button. Items were presented only once during testing. The test duration was on average 30 min. The children were not allowed to take a break since the test was automatized such that the subsequent stimulus began immediately after the previous answer was given. In contrast to other batteries, stimuli were presented in a random order (described in Supplementary Material) to avoid local independence (i.e., contingencies in responses) and artifacts due to fatigue.

### Clinical methodology

#### Sample size calculation

We considered at least 10 participants per observed indicator variable (i.e., the 80 items) as a rule-of-thumb for a lower bound adequate sample size as suggested by Nunnally (1967), totaling at least 800 children. To accurately perform invariance testing, that number was inflated to 1000.

#### Selection of children

For each school, teachers were given instructions on how to randomly select 14 students per grade, from first to fifth, using www.random.org, returning on average 70 children per school.

Teachers, nominated by the school principals, evaluated the children on the MP test. Based on the school's enrollment list for each grade, five working days were allowed for the students' parents or guardians to return informed consent about their selected child's participation in the research. If there was no interest in participating or no return of informed consent, another child was selected to replace them using the same process. This method of random selection without any kind of inclusion/exclusion criteria was used to maximize generalizability and representative sampling of the MP spectrum. For the same reason, no exclusions were made based on teacher or parent report of previous medical or psychological conditions, intellectual disabilities, hearing loss or language impairment.

#### School selection

A stratified random sample of 14 elementary schools was chosen from a pool of Sao Paulo districts and cities where the last author had prior agreement with the Department of Education to collect and conduct research. The cities included were São Paulo, Jacarei, Marilia, and Oscar Bressani. Thirty five percent of the invited schools were private schools in order to provide an adequate sample for invariance testing. The number of private schools was almost twice oversampled compared to official enrollment reports from 2014 (18.6%). The stratified random sample of schools was selected based on the list of schools from the Departments of Education of the four cities; if a school Principal was unwilling to participate, another school was selected.

#### Data collection

A Java program, Armonikos, running offline on Java Virtual Machine (JVM) was installed on a computer in the school. The JVM platform was chosen because it is an independent operating system, and offers scalability to meet future demands. Answers were collected and stored in a local database; IDs were assigned to each computer, to each child, and also to each collection of results (time stamped) to facilitate audit of operator, site, date and machine. The local database files were sent to a centralized server to create a merged dataset. That server was queried to extract consolidated data for analysis. The Armonikos testing procedure is available for research purposes from the corresponding author free of charge.

### Statistical analysis

#### Fitting the model

Confirmatory factor analysis is an important analytical tool to test constructs (also called latent variables, factors or dimensions) underlying sets of observed variables (i.e., items in a questionnaire, set of stimulus within a battery). Normally the items in MP batteries (the stimuli) are dichotomous (i.e., correct or incorrect) and when the items are categorical, CFA is also referred to as item response theory analysis (Takane and De Leeuw, 1987; Du Toit, 2003; Baker and Kim, 2004). CFA is used to validate constructs of psychological scales because CFA accurately estimates underlying latent factors (Embretson, 2004); however these techniques have yet to be transposed into the field of musical perception (MP) and cognition. Since a latent modeling approach has not been used to investigate the constructs underlying previous MP batteries, knowledge of how well the hypothesized models fit the empirical data remains lacking. One study used classical test theory to validate the MBAE in a Brazilian population (Nunes-Silva and Haase, 2012); however, classical test theory does not present a statistical model that permits testing of falsifiable assertions about the properties of a scale (Zimmerman, 1975; Raykov and Marcoulides, 2011; Steyer and Eid, 2012).

The bifactor model has been recently rediscovered as a useful tool to better understand issues regarding the viability and reliability of subscales (Reise, 2012) where the “…general factor *runs through* all the items effectively capturing their shared content with a unifying concept” whereas the specific factors “…account for response variation that is unique or particular to item subsets” Stucky and Edelen (2015). Further advantages of the bifactor model are discussed in Chen et al. (2012). The bifactor model, with its model fit indices, evaluates (a) the unique contribution of the general MP factor and (b) specific factors, to scale each individual on a single trait, but at the same time, to control for the distorting effects of multidimensionality caused by specific item content (Reise et al., 2010). The general factor reflects the battery's target construct, MP, and the seven orthogonal (i.e., not correlated) group factors (also called subscales) represent subdomain constructs based on clusters of items with similar content. For major details about the bifactor model and its derived fit indices that assess viability and reliability of subscales, see Rodriguez et al. (2016a).

Confirmatory factor analysis was used to test the conceptual bifactor model (also known as a general-specific model) *a priori* underlying the 80 items as shown in Figure 1. CFA was chosen to illuminate the latent structure underlying the observed variable, whereas principal components analysis is sometimes used to provide construct validity for MP tasks (Gordon, 1986; Law and Zentner, 2012); however, principal components analysis is better suited to data reduction (Bartholomew, 2004; Borsboom, 2006; Raykov and Marcoulides, 2012) and it would not allow us to evaluate the latent structure or even to test a model.

**Figure 2:**
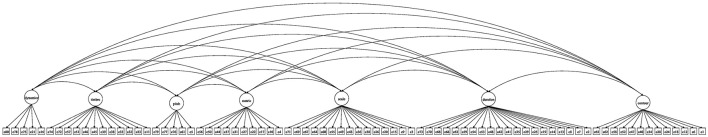
**Diagram representing the seven-correlated factor structure underlying the 80 items of the music perception test**.

**Figure 3:**
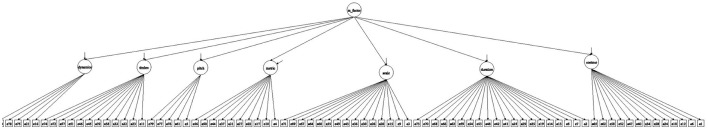
**Diagram representing the second-order model structure underlying the 80 items of the music perception test**.

Using CFA, alternative models about the construct of MP might be conceptually tested as, for example, a correlated-factor model (Figure 2) where the seven factors are all free to be correlated with each other, or even considering a second-order model (Figure 3). where the seven first-order factors would be predicted by a higher-order factor (m-factor). In the latter model, the m-factor would be assumed to have a direct effect on the lower-order factors (e.g., contour, scale, timbre, etc.). Both bifactor and second-order factor models are alternative approaches for representing general constructs comprised of several highly related domains (Chen et al., 2006). However, second-order models do not directly model a common latent variable that runs among the whole set of items (Reise et al., 2010) because it imposes a measurement structure on the correlations between primary factors, attempting to model the correlations between the primary factors instead of directly on the items *per se* (Reise, 2012). Our interest in the bifactor model for MP is justifiable:

1. The bifactor model solution is the least restrictive model when compared to (a) the correlated-factor model, (b) the second-order model, (c) a unidimensional model (all the items loaded onto a single factor). Therefore, these alternative models, which are nested within the bifactor model, significantly degrade that fit. As advised by Yuan and Bentler (2004), it is advisable to consider applying a more restricted nested model only if the least restricted model (in this case the bifactor) is judged to fit the data. In other words, if the bifactor model fits the data, other more restricted models might be tested. The numbers of free parameters in these alternative models, direct indicators of how restrictive the competing models are, range from 240 in the bifactor model, to 181 in the correlated-factor solution, to 167 in the second-order solution, and 160 free parameters in the unidimensional model, which is the most restrictive.
2. Separating the reliable variance of the m-factor (the general MP factor) from the seven specific factors should be conducted exclusively via a bifactor model due to its orthogonality among the specific factors and general factor. The bifactor model can directly examine the strength of the relationship between the domain specific factors and their associated items because the relationship is reflected in the factor loadings, whereas these relationships cannot be directly tested in the second-order model because the domain specific factors are represented by disturbances of the first-order factors (Chen et al., 2006).

To evaluate the goodness of fit of the proposed bifactor model, the following indices were used: chi-square (χ^2^), Confirmatory Fit Indices (CFI), the Tucker-Lewis index (TLI), and root mean square error approximation (RMSEA). The cutoff criteria used to determine the goodness of fit are described as following: chi-square with no statistical significance (>0.05), RMSEA near or less than 0.08 (Little et al., 2013), and CFI and TLI near or greater than 0.9 (Hu and Bentler, 1999). CFI and TLI are penalized under complex models (i.e., multidimensional models with many items per factor), and such models, as proposed here, tend to worsen as the number of variables in the model increases (Kenny and Mccoach, 2003). Accordingly, CFI and TLI's values near to 0.9 were considered a good fit. We used the weighted least square using a diagonal weight matrix with standard errors and mean- and variance-adjusted (WLSMV) estimator. This estimator is the default in Mplus under categorical data (Muthén and Muthén, 2012) and it has been observed that the magnitude of the loadings are more precisely estimated under it (Beauducel and Herzberg, 2006). Due to the cluster structure of the current data (i.e., children nested in schools), the standard errors and chi-square test of the model fit took into account this non-independence using the implementation proposed by Asparouhov (Asparouhov, 2005, 2006). The statistical significance level adopted was 0.05.

### Invariance testing

A common issue recurrent in the psychometric literature but which has yet to be resolved in MP data relates to population heterogeneity and exploration of the stability of the items under different background variables (e.g., sex and age). Invariance testing is a fundamental psychometric procedure to determine if the measurement properties of the items, and of the model, are comparable across important demographic features (e.g., sex, age, race). Invariance testing is pertinent in the context of MP since cultural aspects of MP have been explored using the MBEA, and parts of the original version were shown not to be suitable for evaluating musical abilities across populations with different musical traditions (Paraskevopoulos et al., 2010). In other words, if the researcher or clinician seeks to compare MP across cultures, the items and their underlying factors need to be comparable (invariant). There are different procedures to study the invariance of a given model including multiple indicators, multiple causes (also known as MIMIC also called CFA with covariates Jöreskog and Goldberger, 1975; Muthén, 1989), multi-group CFA, and a more recent method called alignment (Muthén and Asparouhov, 2014). For a review of these procedures and other modern invariance testing techniques see Van De Schoot et al. (2015).

We used MIMIC model to explore the effects of sociodemographic variables (i.e., age, sex, and school type [private vs. public]) on the general MP factor and separate items. This procedure clarifies aspects of measurement invariance and heterogeneity. The former results from inspection of direct relationships between the sociodemographic variables and items that are not mediated by the general MP factor. If significant, this indicates measurement non-invariance due to *differential item functioning* (DIF). Because we have no *a priori* hypothesis about which items might exhibit differential functioning, we used the approach described by Brown (2015) where direct relationships between the sociodemographic variables and the items were fixed at zero; then, upon inspection of modification indices, we freed the effect of sociodemographic variables on the items with the highest modification indices (superior to 4.00) and determined whether this enhanced the model.

Population heterogeneity was explored via relationships between the sociodemographic variables and the general MP factor. If significant, this indicates that the factor means are different for different levels of the sociodemographic variables. For two dichotomous covariates (school type and sex), the heterogeneity effect on the m-factor is given as Cohen's *d* (standardized effect size) where 0.2 to 0.3 is considered a “small” effect, 0.5 a “medium” effect and anything higher than 0.8 a “large” effect (Cohen, 1977). For age, the effect is expressed in terms of a standardized regression coefficient.

### Viability of subscales

The following indices were used to better understand the viability of subscales: (a) coefficient omega (ω; Revelle and Zinbarg, 2009; Mcdonald, 1999), a factor analytical model-based reliability estimate, originating from the work of Jöreskog (1971) estimating the proportion of variance in the observed total scores attributable to all modeled sources of common variance; (b) a coefficient omega hierarchical (ω_h_; Mcdonald, 1999; Zinbarg et al., 2005), model-based reliability index, which judges the degree to which composite scale scores are interpretable as a measure of a single common factor. ω_h is_ was computed by dividing the squared sum of the factor loadings on the general factor by the model estimated variances of total scores; (c) omega subscale (ω_s_), a reliability estimate for a residualized subscale controlling for that part of the reliability due to the general factor (Reise, 2012); and (d) the explained common variance (ECV) defined as the ratio of variance explained by the general factor divided by the variance explained by the general and group factors. Key details about each index, its calculation, and interpretation are described in Rodriguez et al. (2016b).

## Results

### Demographic characteristics

In total, 1006 children were tested, 69.9% of whom were enrolled in public schools, 45% male, with approximately 200 children from each grade 1 through 5. The mean ages and standard deviations (SD) by grade were: first grade (mean = 6.44, *SD* = 0.83), second grade (mean = 7.25, *SD* = 0.63), third grade (mean = 8.37, *SD* = 0.80), fourth grade (mean = 9.35, *SD* = 0.8), and fifth grade (mean = 10.29, *SD* = 0.7). Five out of 14 schools were private schools, representing the non-proportional enrolled students in São Paulo State. Table 1 shows the number of boys and girls across the grades, by type of school (private/public).

**Table 1:** **Sex distribution across grades and type of school**.

**School type**	**Grade**	**Sex**		**Total**
		**Female**	**Male**	
Private	First Grade	32	31	63
	Second Grade	28	31	59
	Third Grade	33	30	63
	Fourth Grade	36	16	52
	Fifth Grade	32	29	61
		161	134	295
Public	First Grade	77	62	139
	Second Grade	72	69	141
	Third Grade	67	63	130
	Fourth Grade	90	71	161
	Fifth Grade	86	54	140
		392	319	711

### Modeling music perception

The goodness of fit for the bifactor model's seven specific factors and general m-factor returned a satisfactory adjusted model: $\chi_{\left(3000\right)}^{2}$ = 3415.408, *p* < 0.001; RMSEA = 0.012 (90% confidence interval [CI] = 0.010 to 0.014), Cfit = 1.000; CFI = 0.931 and TLI = 0.927.

The standardized factor loadings for each item on the m-factor and on specific factors are shown in Table 2, where the items are grouped by the specific factor. Details about the music mode (i.e., tonal, atonal, electronic noise, and others) and paradigm (i.e., same or different) are also identified. Details of the bifactor model, standard errors for each factor loading, and their respective *p*-values are available from the corresponding author. The corresponding musical stimuli descriptions are presented in Supplementary Material.

**Table 2:** **The 80 items' factor loadings under bifactor model on the M−factor and on specific factor**.

**Item**	**Paradigm**	**Musical modus**	**M−factor**	**Contour**	**Duration**	**Scale**	**Metric**	**Pitch**	**Timbre**	**Dynamics**
E1	Different	Tonal	0.506	0.283						
E6	Equal	Tonal	−0.455	−0.294						
E12	Different	Atonal	0.496	0.204						
E18	Equal	Atonal	−0.621	−0.114						
E24	Different	Percussion/Eletronic Noise	0.534	0.161						
E28	Different	Tonal	0.551	0.036						
E34	Different	Atonal	0.619	0.100						
E40	Different	Percussion/Eletronic Noise	0.635	0.111						
E47	Equal	Percussion/Eletronic Noise	−0.629	−0.157						
E52	Equal	Atonal (with eletronic noise)	−0.584	0.192						
E58	Different	Atonal (with eletronic noise)	0.616	−0.254						
E62	Different	Atonal (with eletronic noise)	0.584	−0.270						
E65	Equal	Atonal (with eletronic noise)	−0.594	0.309						
E2	Equal	Percussion/Eletronic Noise	−0.443		0.348					
E7	Different	Simple sound comparison	0.422		0.384					
E8	Different	Percussion/Eletronic Noise	0.490		0.291					
E13	Different	Simple sound comparison	0.468		0.201					
E14	Different	Percussion/Eletronic Noise	0.517		0.178					
E19	Equal	Percussion/Eletronic Noise	−0.482		0.066					
E25	Different	Percussion/Eletronic Noise	0.576		0.207					
E29	Equal	Percussion/Eletronic Noise	−0.513		0.178					
E35	Different	Percussion/Eletronic Noise	0.497		−0.084					
E41	Equal	Simple sound comparison	−0.507		0.245					
E42	Different	Percussion/Eletronic Noise	0.599		0.274					
E48	Different	Atonal	0.621		−0.002					
E53	Different	Simple sound comparison	0.493		0.437					
E54	Equal	Tonal	−0.486		0.417					
E59	Equal	Tonal	−0.595		0.263					
E63	Equal	Atonal	−0.519		0.208					
E66	Different	Atonal	0.612		−0.044					
E68	Equal	Modal	−0.552		0.389					
E70	Different	Tonal	0.593		−0.020					
E73	Equal	Simple sound comparison	−0.444		0.258					
E3	Equal	Tonal	−0.431			−0.294				
E9	Different	Modal	0.481			0.338				
E15	Different	Modal	0.516			0.252				
E20	Different	Tonal	0.574			0.263				
E26	Equal	Modal	−0.587			−0.333				
E30	Different	Tonal	0.585			0.439				
E36	Equal	Modal	−0.616			−0.055				
E43	Different	Modal	0.601			−0.084				
E49	Equal	Tonal	−0.594			−0.171				
E55	Different	Modal	0.649			0.161				
E60	Equal	Modal	−0.582			−0.053				
E64	Different	Tonal	0.660			−0.054				
E67	Different	Modal	0.570			0.166				
E69	Different	Atonal	0.595			0.069				
E71	Different	Atonal	0.657			−0.288				
E4	Different	Percussion/Eletronic Noise	0.518				0.364			
E10	Equal	Percussion/Eletronic Noise	−0.506				0.128			
E17	Equal	Atonal	−0.532				0.076			
E22	Different	Atonal	0.588				−0.037			
E27	Different	Percussion/Eletronic Noise	0.570				0.317			
E31	Equal	Percussion/Eletronic Noise	−0.597				0.080			
E37	Different	Tonal	0.495				−0.059			
E44	Equal	Tonal	−0.598				0.448			
E50	Equal	Tonal	−0.594				0.445			
E56	Different	Modal	0.565				0.215			
E5	Different	Simple sound comparison	0.348					0.285		
E61	Different	Simple sound comparison	0.517					−0.391		
E76	Equal	Simple sound comparison	−0.488					0.271		
E77	Different	Simple sound comparison	0.407					0.132		
E79	Different	Simple sound comparison	0.556					−0.426		
E11	Different	Modal	0.350						0.453	
E23	Different	Atonal	0.393						0.541	
E32	Different	Percussion/Eletronic Noise	0.352						0.714	
E33	Equal	Modal	−0.574						0.003	
E38	Different	Simple sound comparison	0.188						0.760	
E39	Different	Percussion/Eletronic Noise	0.400						0.293	
E45	Different	Simple sound comparison	0.213						0.742	
E46	Equal	Percussion/Eletronic Noise	−0.498						−0.032	
E51	Different	Atonal (with eletronic noise)	0.598						−0.011	
E57	Equal	Percussion/Eletronic Noise	−0.541						0.040	
E72	Different	Atonal	0.396						0.456	
E74	Different	Tonal	0.616						0.351	
E16	Equal	Simple sound comparison	−0.463							0.170
E21	Different	Simple sound comparison	0.427							0.461
E75	Different	Simple sound comparison	0.489							0.444
E78	Different	Simple sound comparison	0.390							0.486
E80	Different	Simple sound comparison	0.551							0.434

Standardized factor loadings represent the degree to which each item is associated with its underlying factor; values closer to 1 represent stronger correlations with the underlying factor. A negative correlation indicates that the item is inversely correlated with the underlying factor. The “same” items (i.e., two identical sequences of sounds) loaded onto the m-factor with negative factor loadings. The factor loadings onto the m-factor are higher than those onto the specific factors.

### Viability of the five subscales

The following indices were derived from the bifactor model: ω = 0.977, ω_H_ = 0.938, and EVC = 0.754. From ω_H_, we found that 93.80% the variance in the unit-weighted total scores could be attributed to the differences between participants in the general MP factor. The square root of ω_H_ (0.968) indicates an excellent correlation between the general factor and the observed total scores. The reliabilities of the five specific factors [calculated as ω_(s)_] were very low when controlling for reliability of the general MP factor: ω_(s)Contour_ = 0.088, ω_(s)Duration_ = 0.142, ω_(s)Meter_ = 0.112, ω_(s)Timbre_ = 0.367, and ω_(s)Loudness_ = 0.309, ω_(s)Scale_ = 0.097, ω_(s)Pitch_ = 0.205.

### DIF and heterogeneity population

None of the 80 items showed DIF. It can be concluded that girls and boys with the same level of the m-factor do not differ in their likelihood to respond to any given stimulus correctly. Similarly, no DIF was observed for children enrolled at public vs. private schools, or based on age. Children of different ages, or in different types of schools, who have the same amount of m-factor do not have a higher likelihood of responding correctly to any given item.

Boys showed a higher mean for the m-factor as compared to girls, but this difference was very small in terms of magnitude (*d* = 0.122, *p* = 0.033). Children from public schools had moderately lower values of m-factor as compared to those enrolled in private schools (*d* = −0.416, *p* < 0.001). No effect of age (β = 0.052, *p* = 0.099) was observed; therefore, there is no evidence to suggest that age is associated with MP achievement.

Information curve for all items and latent trait distribution for the general factor.

**Figure 4:**
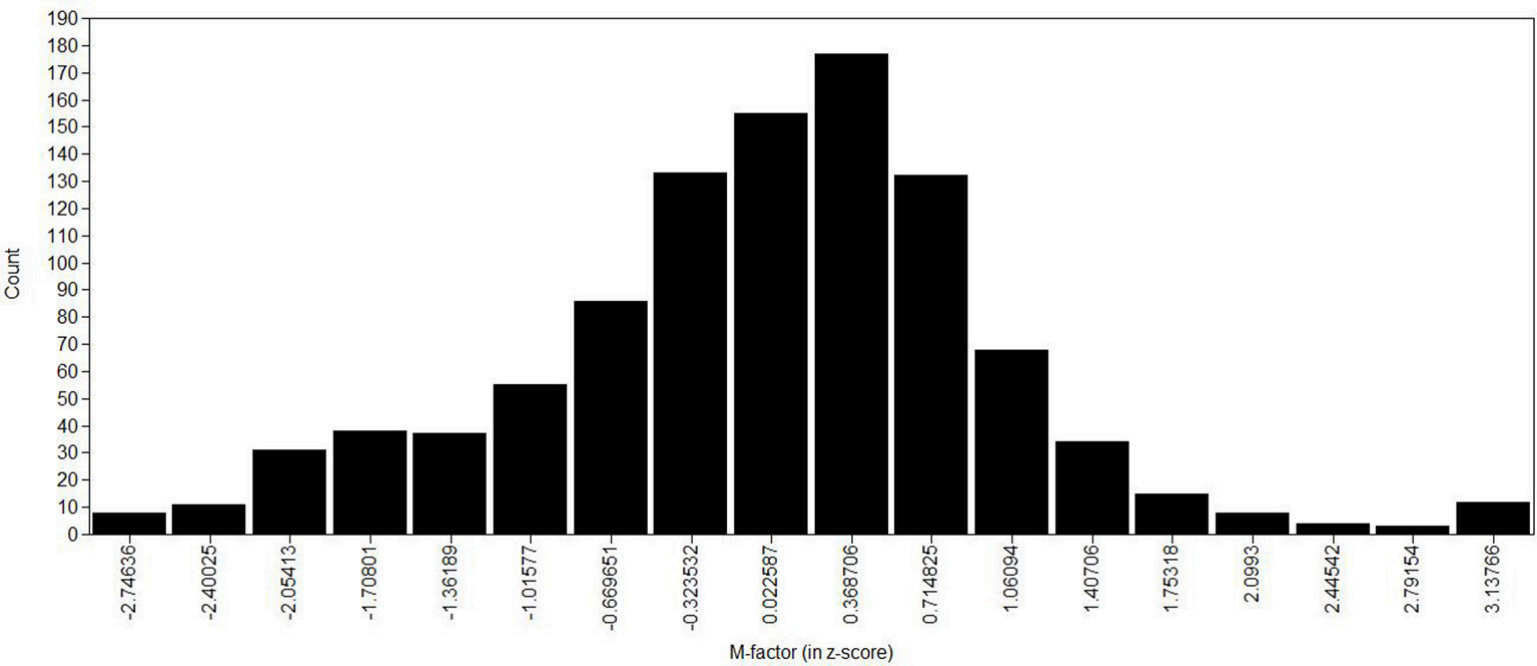
**Histogram of the music perception factor (M-factor) distribution in z-score**.

**Figure 5:**
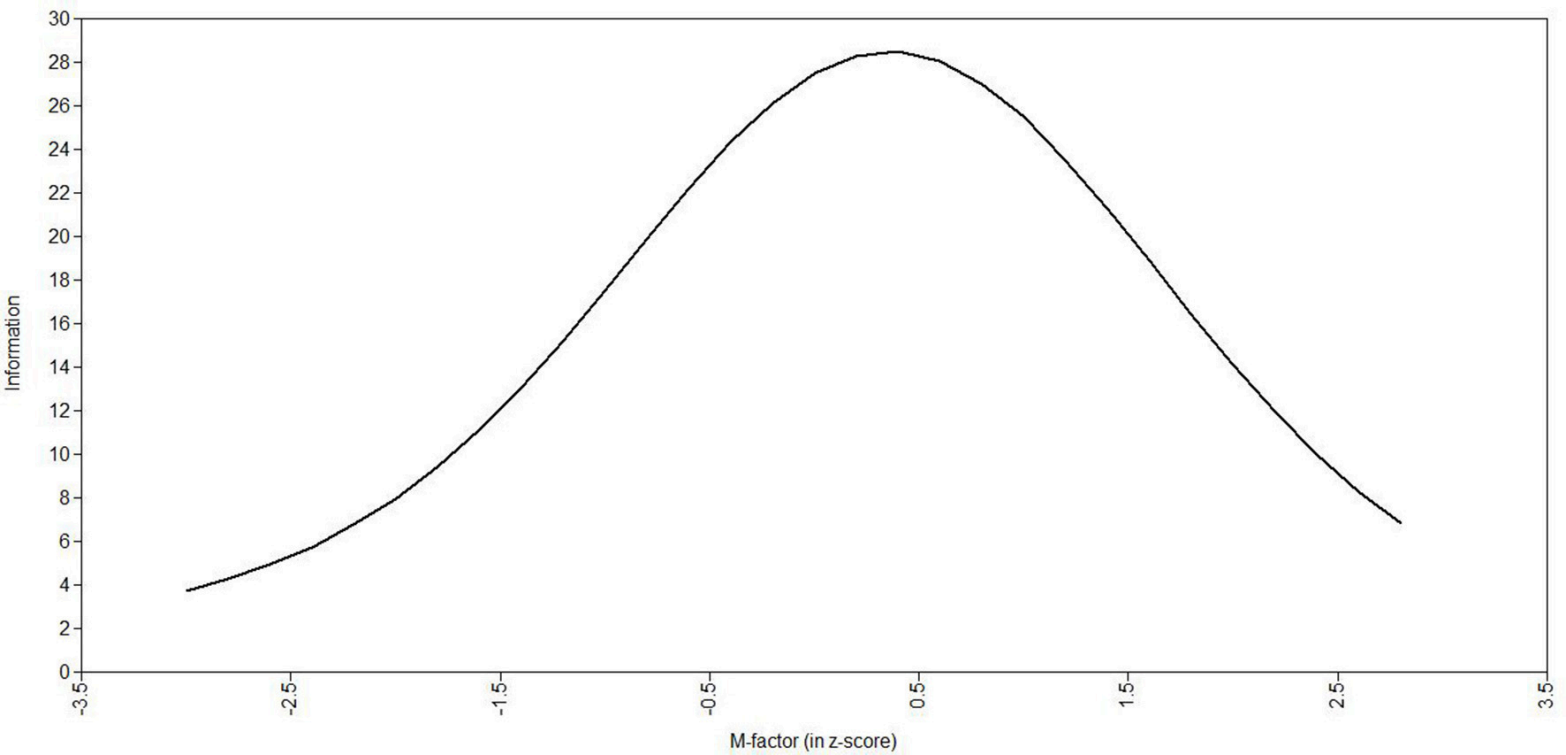
**Total information curve for the music perception factor (M-factor)**.

The distribution of the m-factor (histogram) is shown in Figure 4, demonstrating a normal-like distribution (skewness = −0.062 and kurtosis = 0.970), and the total information curve for the m-factor is presented in Figure 5. The total information curve shows a peak around zero (the mean in the z-score scale), indicating that the 80 items have optimal precision in children with an average amount of MP skill, and less precision in assessing very skilled or very impaired children (greater than 2 standard deviations from the norm). In this figure, the y-axis shows information (precision) of the m-factor (not a probability function to infer normality).

## Discussion

### In support of an *m-factor*

We find strong evidence that the items within each of the seven specific factors converge to inform a general (MP) factor, the *m-factor*. Statistically, the set of 80 items encompassing eight domains (seven specific factors + one general factor) returned excellent fit indices. Musically, the compositional hybrid model used herein extends the content of available scales, balancing items based on the tonal paradigm of the so-called *common-practice period* with two others present in Western music: the modal paradigm and post-tonality. The latter is not a paradigm *per se*, but rather a way to organize musical parameters (i.e., pitch, duration/rhythm, intensity, timbre) in a way that contradicts tonality. These elements of musical language beyond tonality (Boucourechliev, 1993) originate from the musical avant-garde (Griffiths, 2011). Hence, the m-factor offers a more flexible exploration of musical possibilities that does not rely on the assumption that the tonal system is musically universal, allowing assessment of musical understanding beyond a narrowly defined conception of Western tonal music. Although the idea of a general music factor had been described in Law and Zentner (2012) alluding to Charles Spearman's g-factor for intelligence, formal procedures (i.e., bifactor modeling) to evaluate if the data fit to such model had not previously been conducted. Moreover, as in the case of the g-factor, the distribution of the m-factor across a random sample of children was normal.

### A lack of support for subscale viability

Through the bifactor model, we observed that the viabilities and reliabilities of MP subscales were poor. Moreover, when ω_H_ (0.938) is compared with ω (0.977), almost all of the reliable variance in total scores (0.938/0.977 = 0.96) can be attributed to the general factor, which reflects individual differences in MP taken as a whole. Only 3.9% (the difference between 0.977 and 0.938) of the reliable variance in total scores can be attributed to the dimensionality associated with the specific domains. Only 2.3% (i.e., the difference between 1.000 and 0.977) of the estimated variance is due to random error. It is important to stress that although the data advocate for parceling analytically (to recreate the m-factor at a latent level), they do not support reporting at the level of the subscales, because the subscales are not reliable; instead, it is advisable to report only the general m-factor. Summing the number of 80 items answered correctly can be interpreted as an essentially unidimensional reflection of MP, regardless of the multidimensionality evident in the data. In other words, the *m-factor* is robustly reliable even though it is a multidimensional construct, and the specific subdomains displayed weak viability beyond the general MP factor.

This lack of subscale viability is consistent with the findings from bifactor models applied to other areas of child evaluation (Jovanović, 2015; Wagner et al., 2016), and applied to 50 other different scales assessing various aspects of psychopathology and personality (Rodriguez et al., 2016a). From those studies, two main results have arisen: (1) although all measures had been assumed to be multidimensional, unit-weighted total scores overwhelmingly reflected variance due to a single latent variable; and (2) unit-weighted subscale scores mostly reflected the general trait, not specific dimensions (Rodriguez et al. (2016a).

In common practice, findings of multidimensionality are often considered to be a sufficient justification for reporting subscale scores. There are several important concerns, however, with this practice (Reise et al., 2013). Moreover, even in the presence of multidimensionality, the use of total scale scores can be justified (Gustafsson and Åberg-Bengtsson, 2010), whereas findings of multidimensionality do not guarantee that subscales can provide meaningful and reliable information about subdomains that is unique from the general construct.

Despite our lack of evidence supporting subscale viability and reliability, MP subscales have been useful in several different contexts. For instance, the MBEA melodic discrimination ability subscale provided a way of finding out treatment development and cognitive remediation in schizophrenia (Kantrowitz et al., 2014) and the domain of rhythm perception is clearly associated with prosody perception (Hausen et al., 2013). Evaluating the subscale viability of these instruments may help to determine if those correlations are truly subscale-specific.

### Item level features

Given the large sample size, modeling at the item-level was viable in this study. Tools measuring MP and its components tend to use parcels to represent the domains, where the achievement in a given domain is a composite (normally a sum) of the correctly endorsed items. In this procedure, each parcel is treated as the observed indicator rather than evaluating the items individually. When properly modeled, item-level analyses and parcel-level analyses should generally converge on the same centroid (Little et al., 1999). For our battery, we offer evidence in favor of parceling. Researchers interested in using our model for MP at a latent level (i.e., distinguishing common from residual variances) can use a facet–representative parceling procedure for the seven specific factors, even with small sample sizes (Little et al., 2002). By taking the average of the correct items that were assigned to each parcel, the researcher can model a unidimensional trait via confirmatory factor analysis, having seven parcels as the items. We recommend using the average of correct answers per parceling due to the unbalanced number of items per parcel. This procedure will reduce the complexity of the model making the analysis more viable than a full item-level decomposition.

We observed that items under an equal paradigm (pairs of stimuli exhibiting no difference) were negatively related to the m-factor while different items (pair of stimuli where there are differences) were positively related to the m-factor. This indicates a methodological effect on the way the different types of items capture MP skills. Because the other available MP batteries did not investigate features at item-level, inverse factor loading patterns have not yet been described, partially due to noise introduced by parceling procedures adopted in the interpretation of the available MP batteries. Future psychometric investigations might incorporate this new source of variance (the paradigm) into a specific type of confirmatory model called “multitrait-multimethod,” where variance due to paradigm (the multimethod part of the model) and variance due to the seven domains (the multitrait part) are modeled. Thus, CFA offers multiple possibilities for evaluating the psychometric features of MP models and their fit to empirical data. Our decision regarding how to group and test the items was based on the traditional organization of MP batteries (e.g., pitch, scale, meter and so on); however, many other factor structures could be tested. For instance, given 80 stimuli, many of which extend beyond the diatonic paradigm, completely novel specific factors beyond those traditionally conceptualized could be constructed by grouping items based on musical modes (tonal, atonal, modal, electronic noise). These alternative models, if found to fit the data, might allow exploration of novel MP features, particularly if novel subscales are found to be reliable under a bifactor model.

### Stability of the measurement

To our knowledge, invariance testing has not been described in the MP literature. In the present study, we found that none of the items showed differential item functioning regarding age, sex, or type of school. In other words, none of the 80 items have a different probability of being answered correctly by children of different ages, sexes, and school type. Thus, the items are stable indicators of MP. Previously, different hypotheses have been raised in terms of comparing cultures on their MP skills (Zatorre, 2016) and language background (Peretz et al., 2013). However, without precise information regarding batteries designed to measure MP and their invariance features in those target groups, results may be biased due to differential item functioning. In that case, a given musical task might be answered differently between groups (i.e., two groups perceive a given musical paradigm differently given the same amount of MP trait).

### Musical heterogeneity in the population

The present study reports that children enrolled in public schools exhibited a lower amount of m-factor (with a moderate effect size). In contrast, only a very small effect of sex on m-factor was observed–one that would require a similarly large sample size to replicate. Even in this very large sample size including a range of ages, there was no evidence that the m-factor was correlated with age. This contrasts with Peretz et al. (2013), who found a small correlation between the Montreal Battery of Evaluation of Musical Abilities (MBEMA) with age (Pearson's r varying from 0.29 for untrained children to 0.31 for musically trained children) in a population from 6 to 8 years old). Regarding our lack of correlation with age, it is important to emphasize that this is a cross-sectional study. Therefore, inferences regarding the development of MP across childhood are not possible. Moreover, because this is the first study to evaluate invariance across different ages, the finding cannot be compared reliably with studies using other batteries which may be susceptible to differential item functioning based on age. Ideally, longitudinal studies would be necessary to first evaluate the stability of MP across age and then to establish growth trajectories of MP development.

A total information curve for the m-factor showed that the 80 items measure MP most accurately among children with average skills (peak of information at zero on the Z-scale). Therefore, the m-factor can be considered optimal for use with averagely skilled children, and consequently our battery may not optimally measure MP among children with amusia or with exceptional musical skills. Children with congenital amusia are “…unable to recognize well-known tunes in the absence of lyrics, and they have difficulty differentiating melodies on the basis of pitch cues alone, despite having normal hearing, speech, and intellectual ability, and ample opportunity for musical exposure” (Peretz et al., 2013). To optimize assessment of such a poorly skilled population, it would be necessary to know two parameters obtained via confirmatory factor analysis: each item's factor loading and threshold. To identify children with amusia, items exhibiting very low thresholds and with high factor loadings would be needed. Previously, the available batteries to evaluate MP have not reported these data at item-level, and therefore how the constituent items might behave psychometrically in extreme populations remains to be formally tested.

## Limitations

As a potential limitation, only basic socio-demographic features were collected for this study, which aimed to validate the battery and describe its underlying psychometric features. Therefore, information regarding the distribution of IQ, hearing acuity and language impairment were not ascertained; however, since the m-factor was normally distributed it is unlikely that these factors would have introduced significant skewness, and because this large sample was obtained via a true random algorithm selection, the findings are likely to generalize to school-age children in Sao Paulo with their expected distributions of neuropsychological, developmental, and behavioral attributes.

## Conclusion

The present multidimensional battery offers a reliable measure of the m-factor, a new universal non-verbal measure of auditory stimulus apprehension stable across sex, grade at school, and type of school, suitable to study the underlying neurobiology of music perception, the etiology of speech and language disorders, and innate determinants of musicality.

## Author contributions

CB composed the 80 stimulus for the music perception battery. GB, AL, and BI guided CB with the music structure and theory regarding how the stimulus should be composed. WS, AJ, and SM gave insights in the manuscript's writing on the topic of children development and its relation to music perception and language skills. HC run all the analysis, planed the survey, trained the 14 teachers to assess the children. HC, WS, TL, and GP helped in the description of the statistical analysis giving insights about the parceling procedure and bifactor model structure.

## Funding

We are thankful to São Paulo Research Foundation (FAPESP grant number 2014/06662-8 and 2016/50195-0) and CAPES (process number 23038.009191/2013-76, AUXPE n° 0374/2016).

### Conflict of interest statement

The authors declare that the research was conducted in the absence of any commercial or financial relationships that could be construed as a potential conflict of interest. The reviewer KI and handling Editor declared their shared affiliation, and the handling Editor states that the process nevertheless met the standards of a fair and objective review.
