# Supplementary material for: Assessing Music Perception in Young Children: Evidence for and Psychometric Features of the M-Factor
Source: Front Neurosci. 2017 Jan 24;11:18. doi: 10.3389/fnins.2017.00018 (PMC5258735; doi:10.3389/fnins.2017.00018)

Sound stimuli

1 different

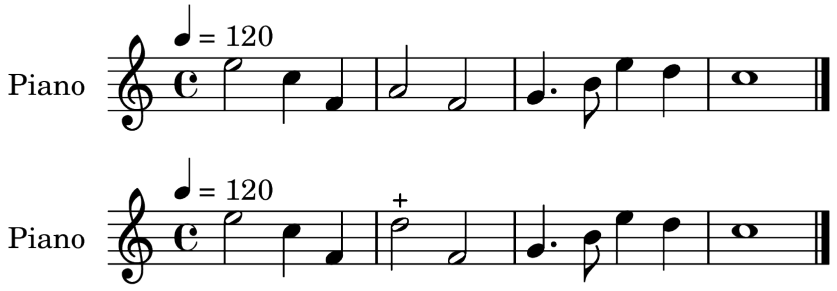

2 equal

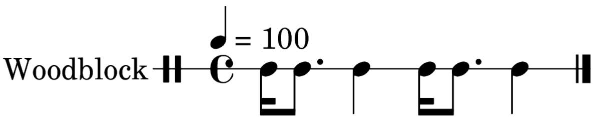

3 equal

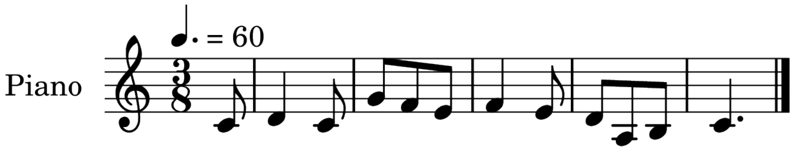

4 different

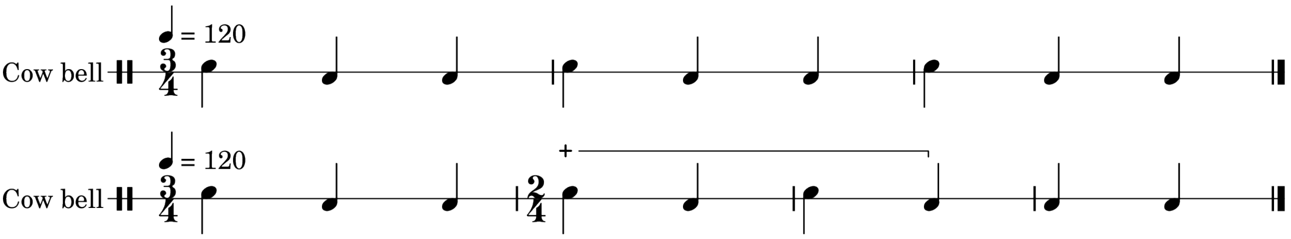

5 different

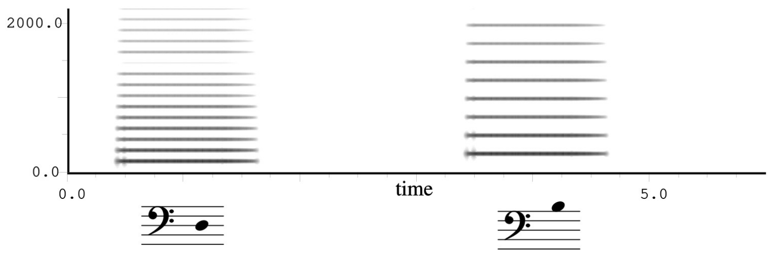

6 equal

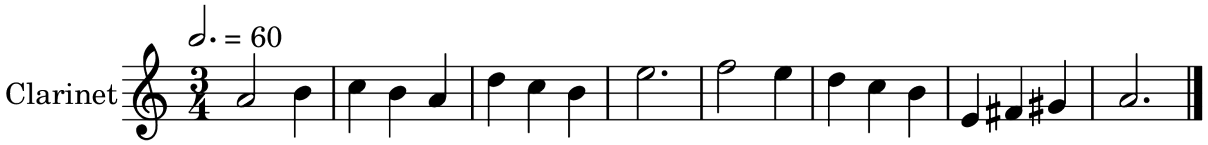

7 different

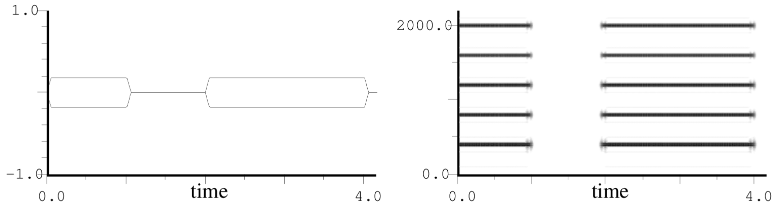

8 different

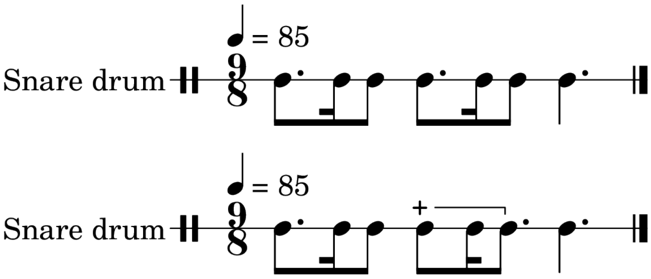

9 different

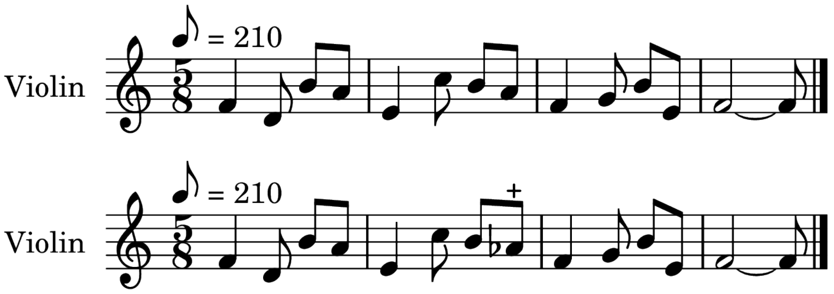

10 equal

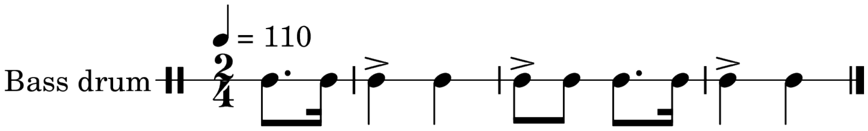

11 different

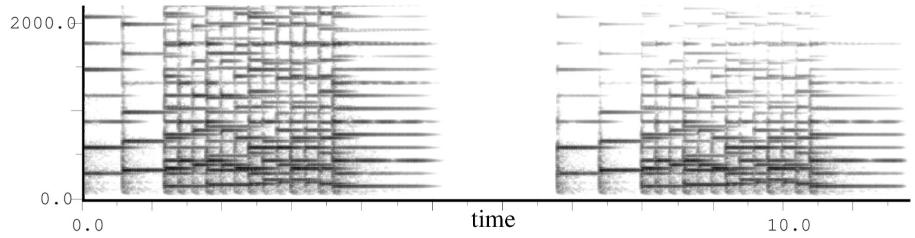

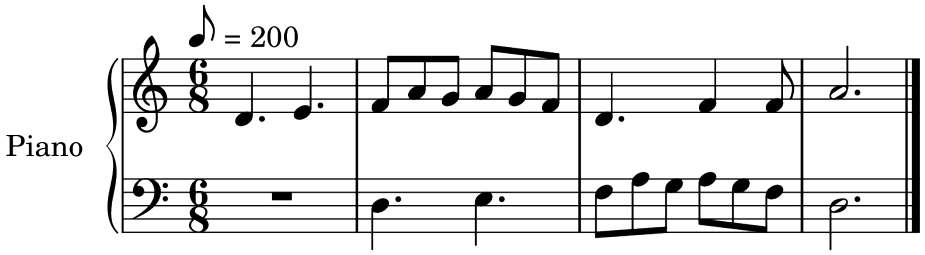

(2nd time with added filters)

12 different

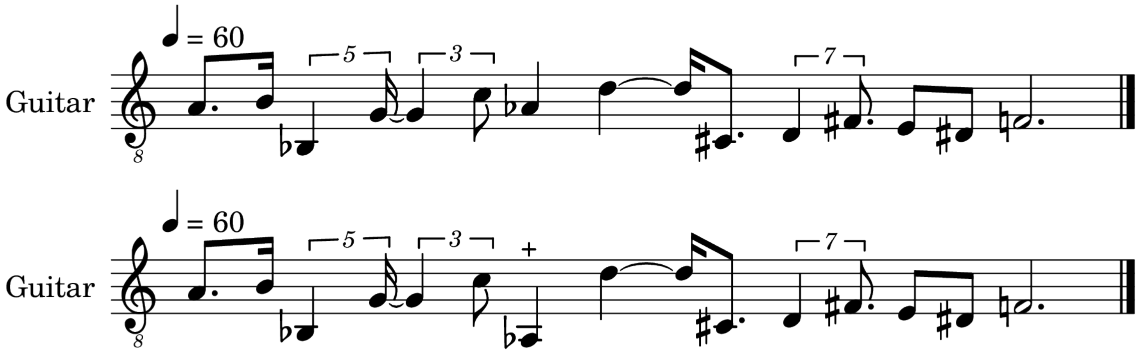

13 different

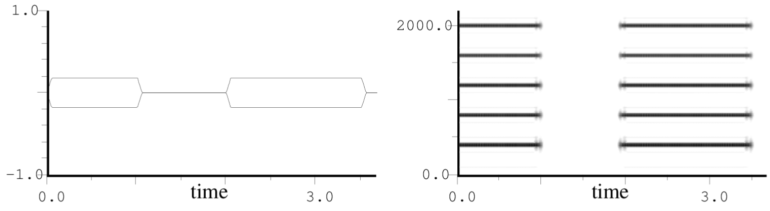

14 different

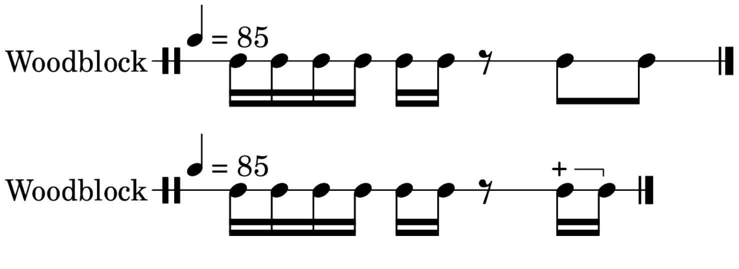

15 different

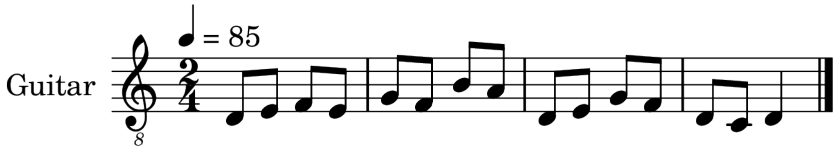

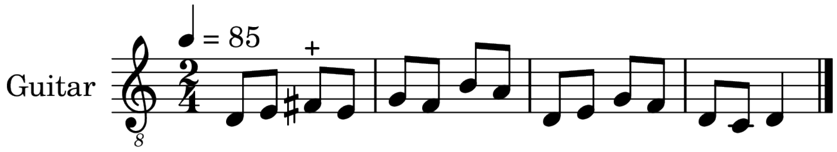

16 different

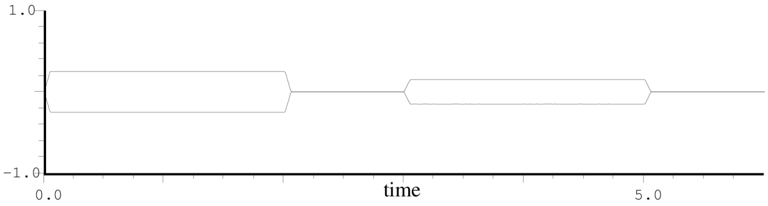

17 equal

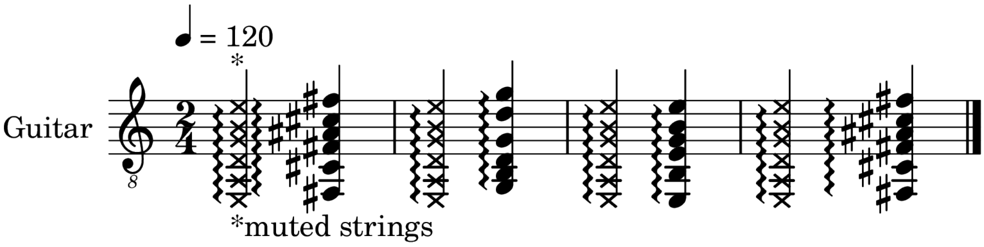

18 equal

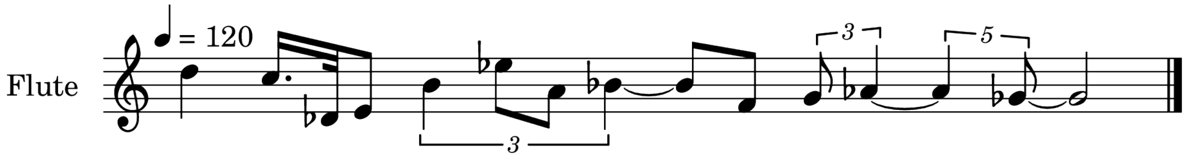

19 equal

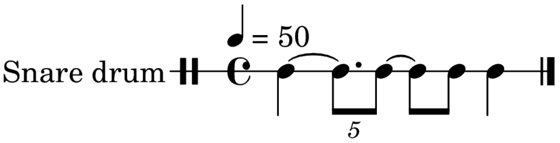

20 different

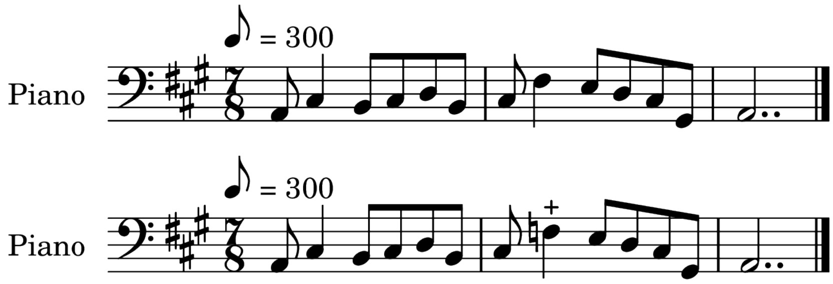

21 different

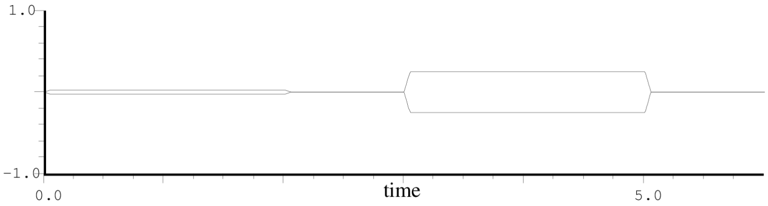

22 different

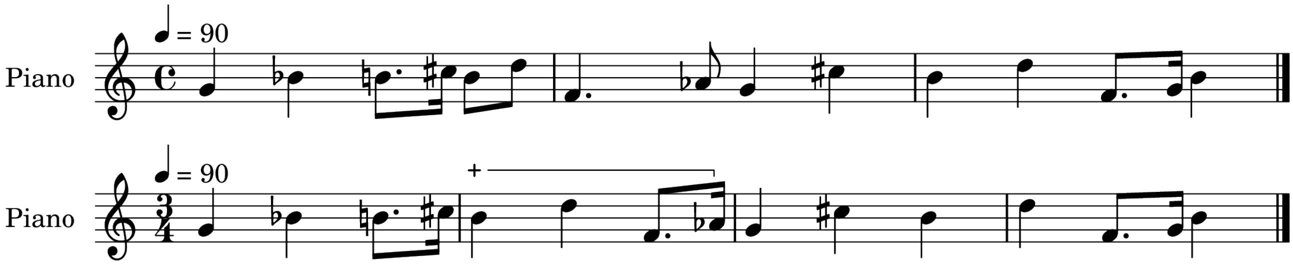

23 different

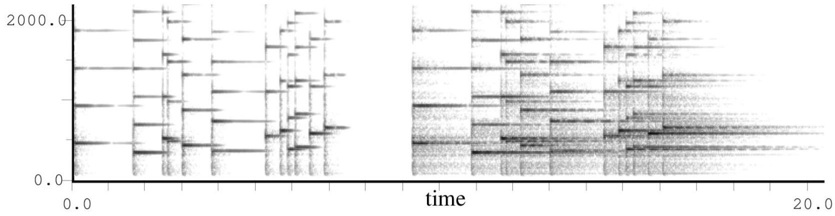

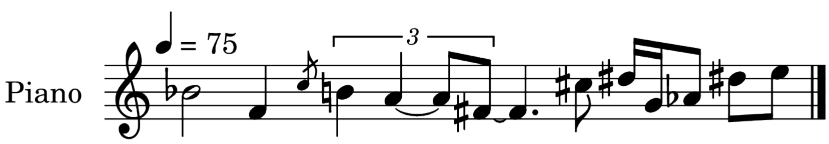

(2nd time with added reverb)

24 different

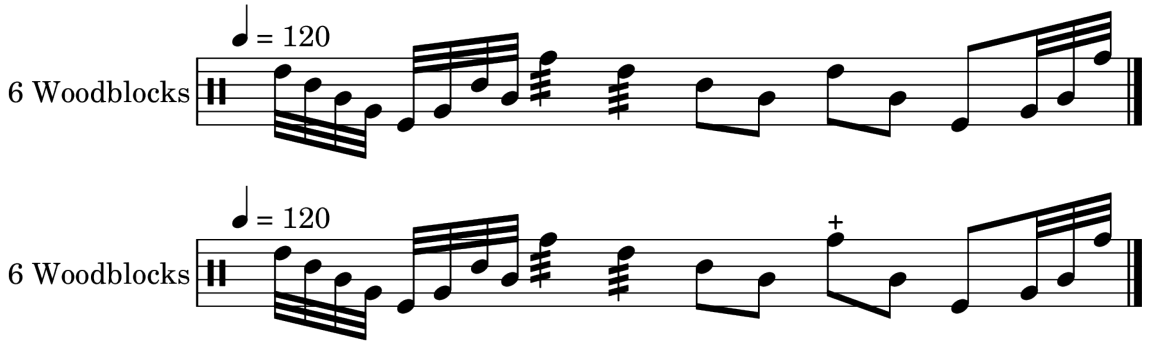

25 different

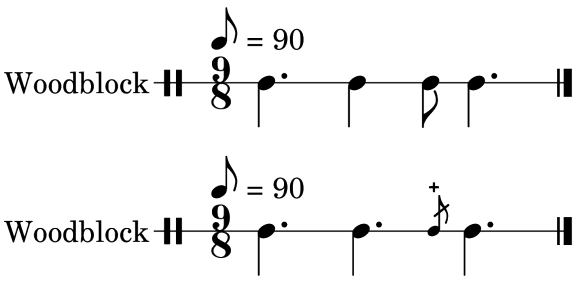

26 equal

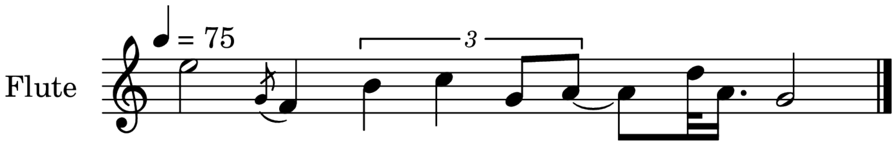

27 different

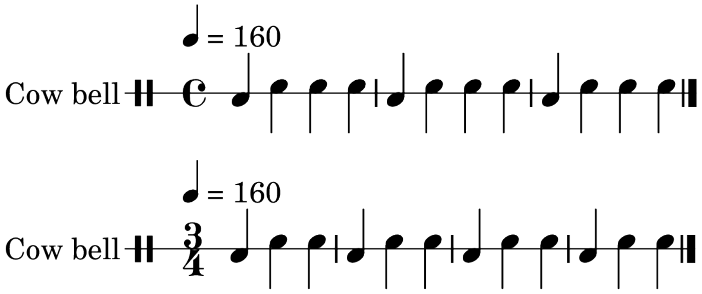

28 different

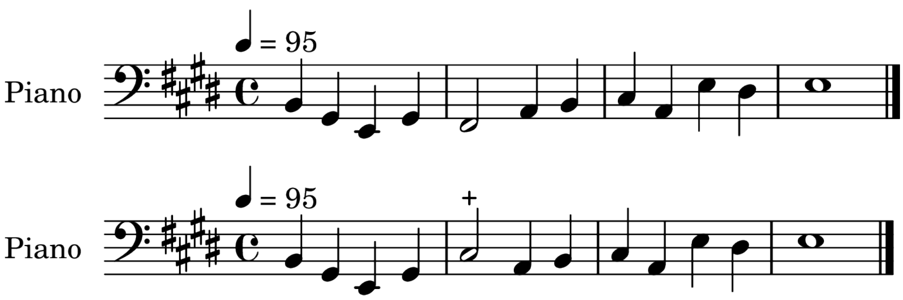

29 equal

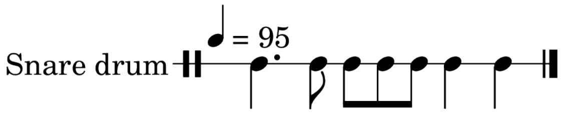

30 different

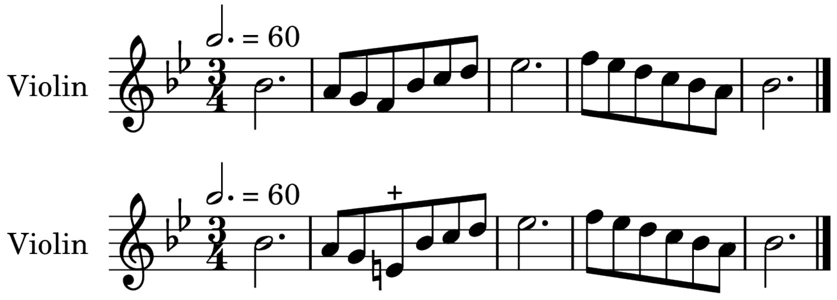

31 equal

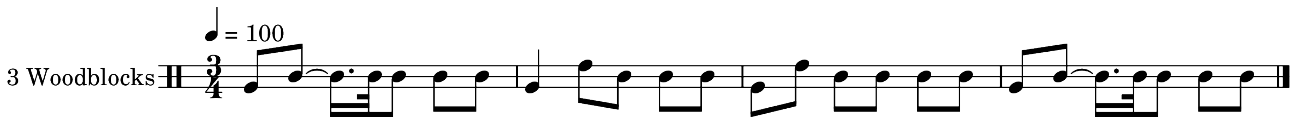

32 different

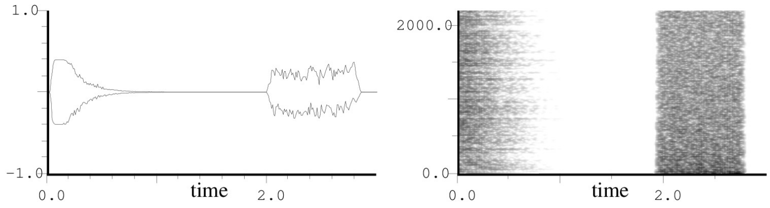

33 equal

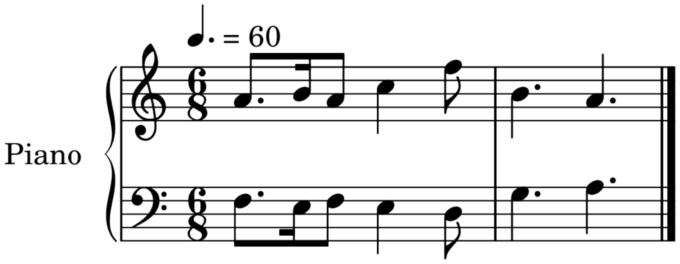

34 different

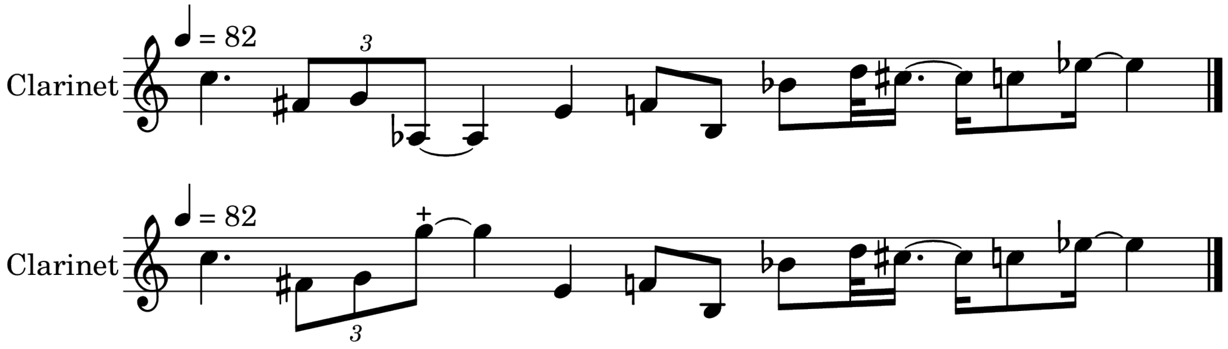

35 different

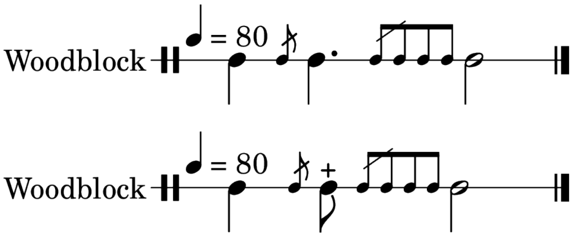

36 equal

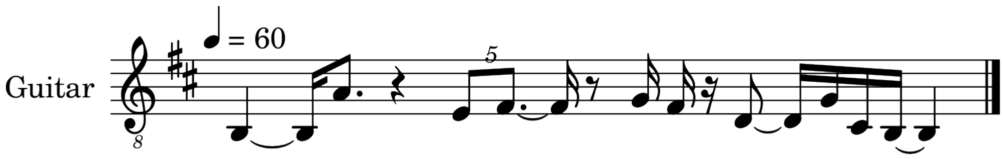

37 different

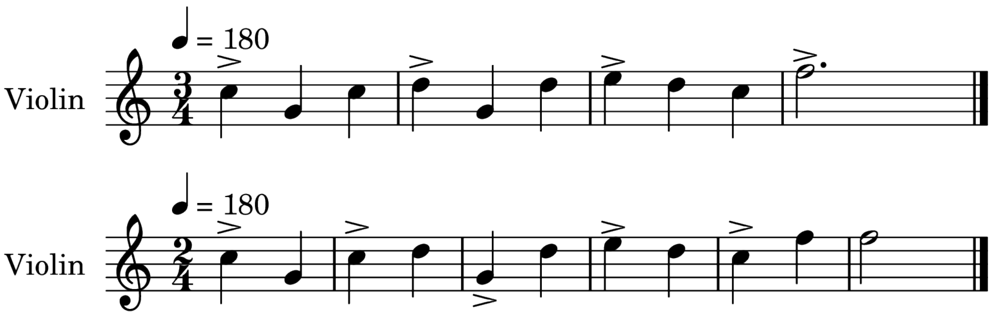

38 different

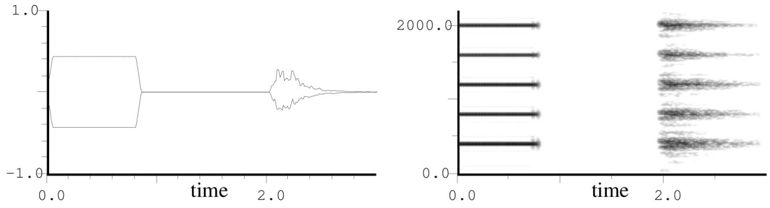

39 different

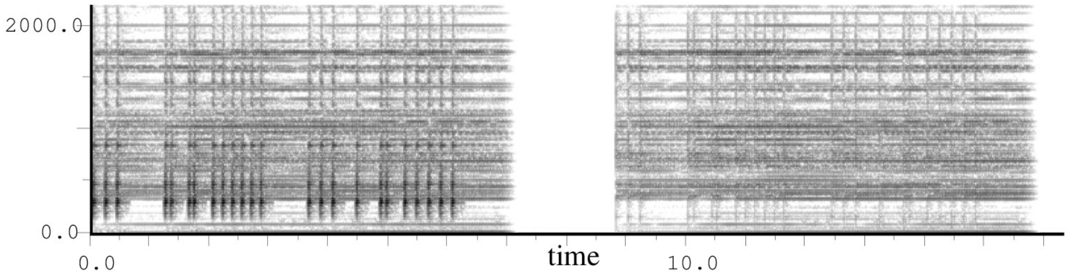

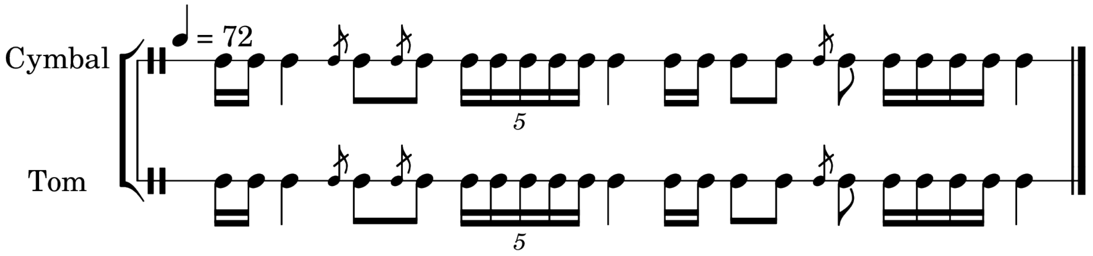

(2nd time Cymbal solo)

40 different

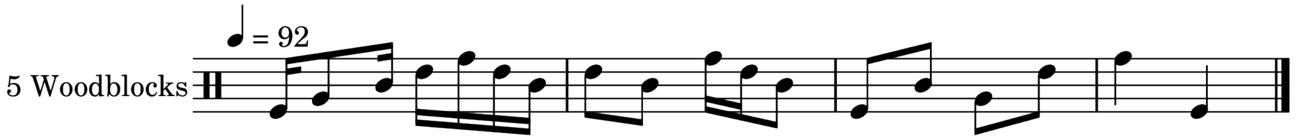

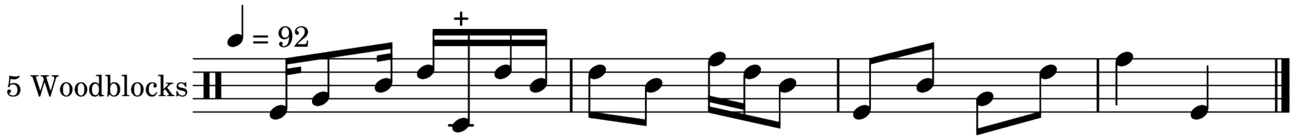

41 equal

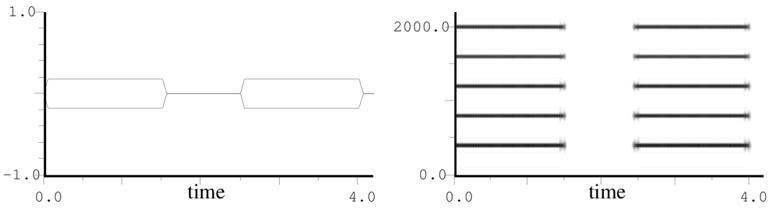

42 different

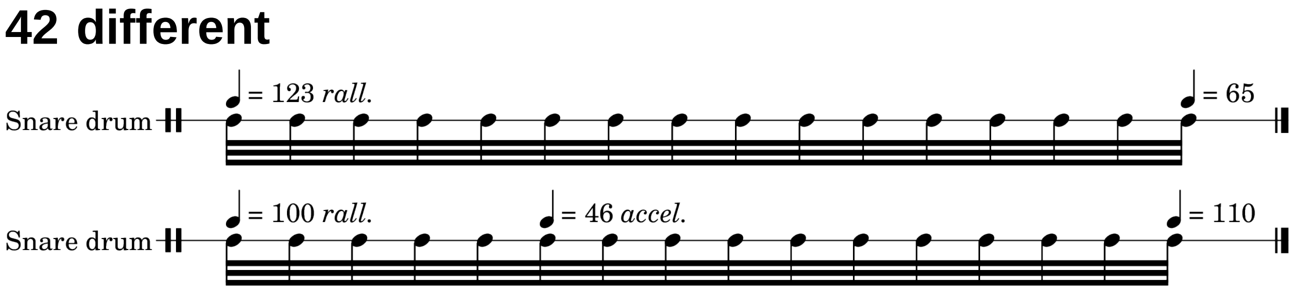

43 different

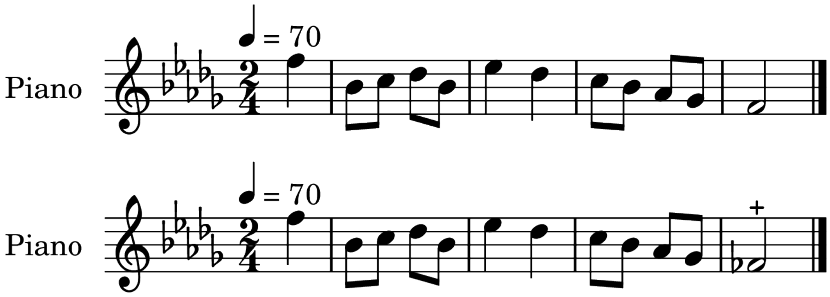

44 equal

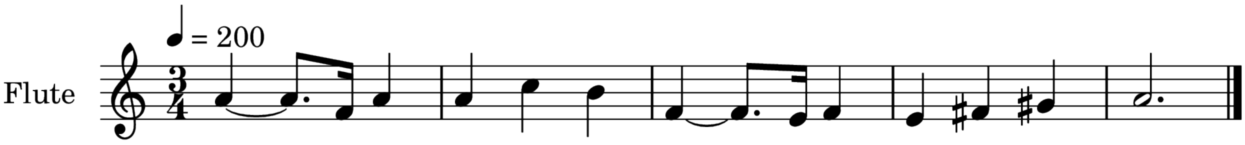

45 different

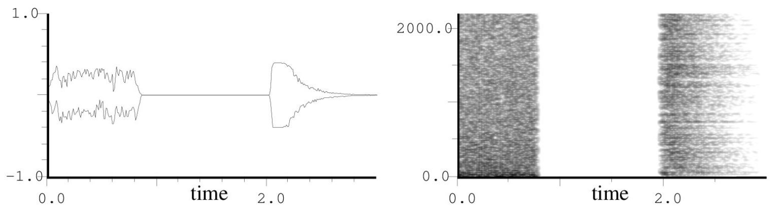

46 equal

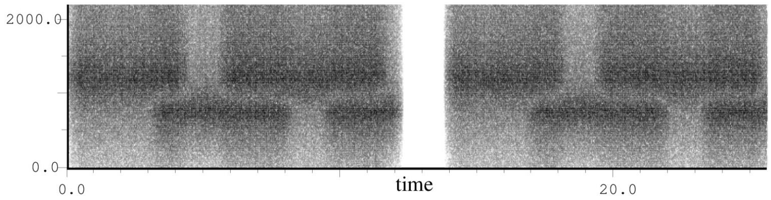

47 equal

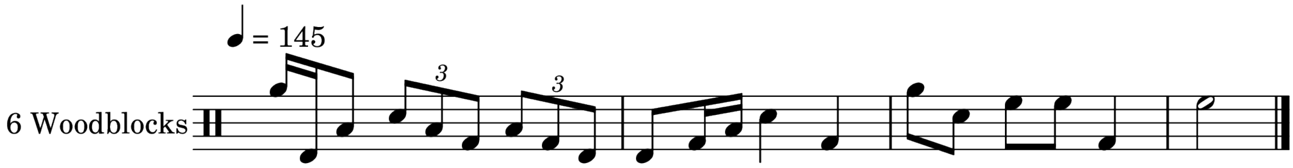

48 different

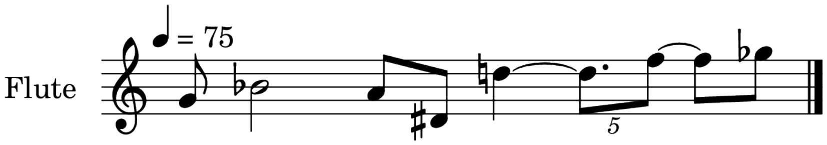

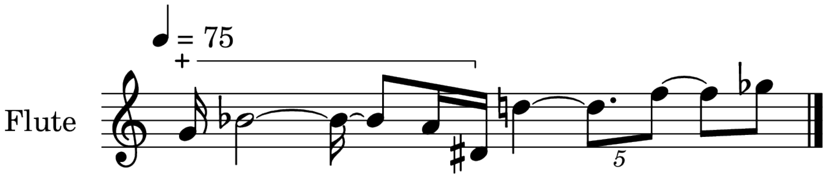

49 equal

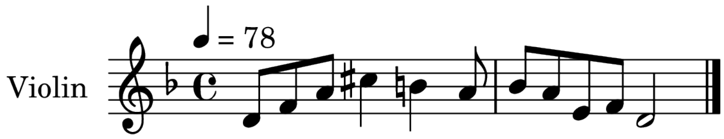

50 equal

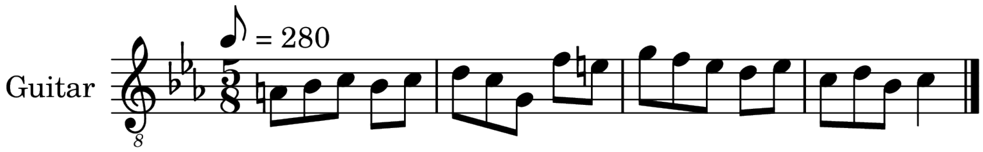

51 different

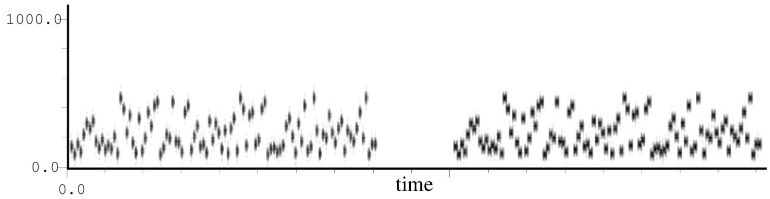

52 equal

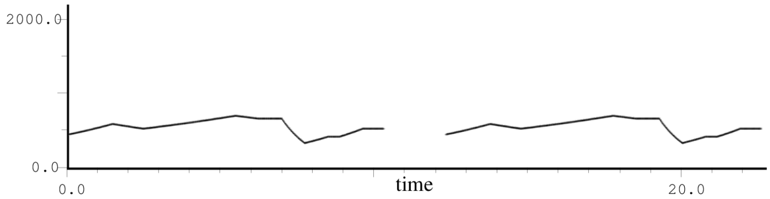

53 different

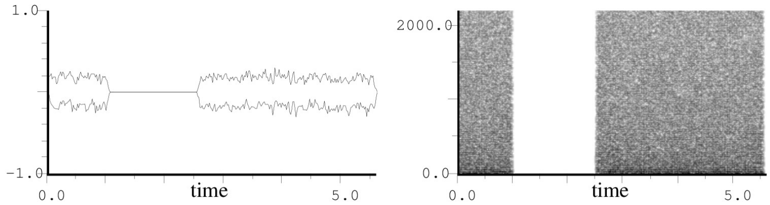

54 equal

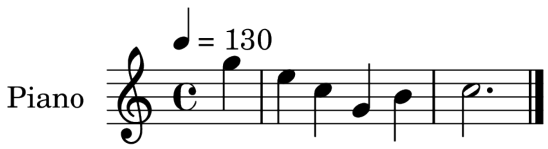

55 different

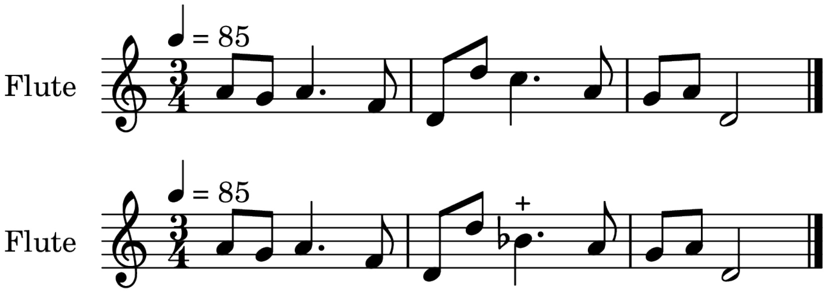

56 different

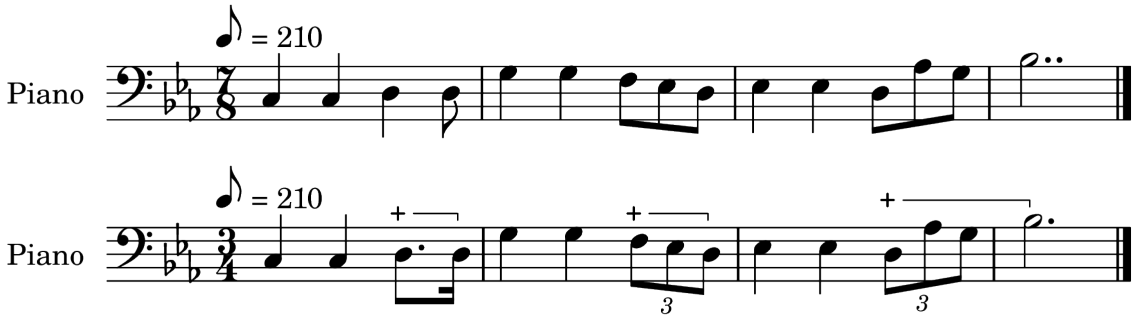

57 equal

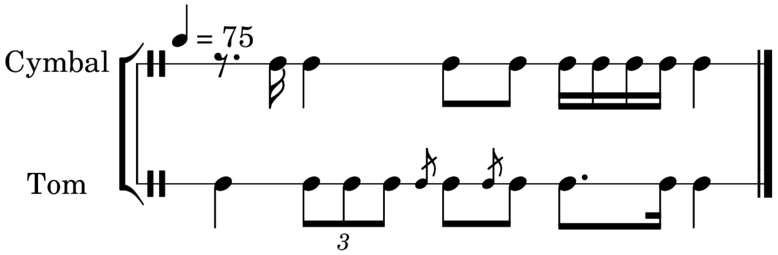

58 different

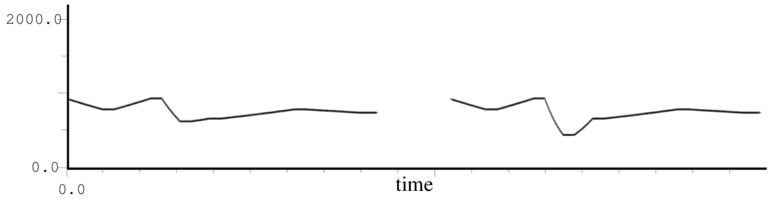

59 equal

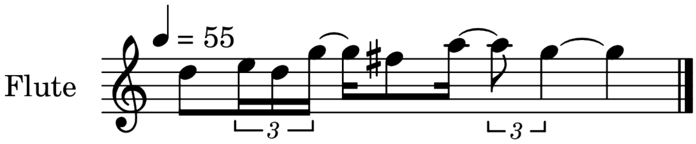

60 equal

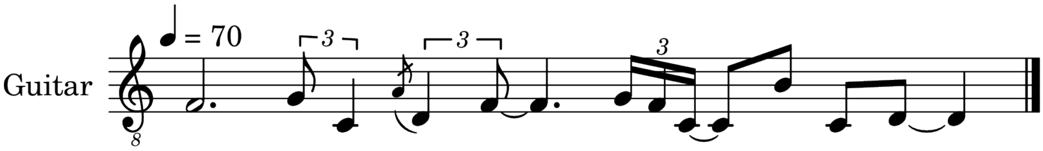

61 different

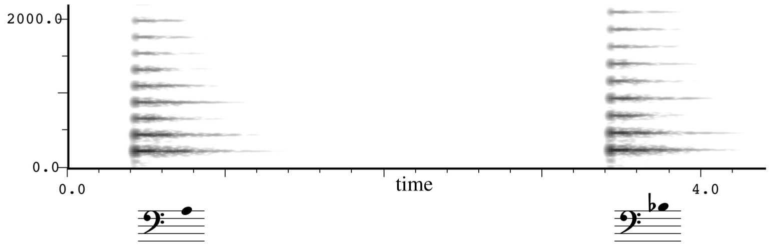

62 different

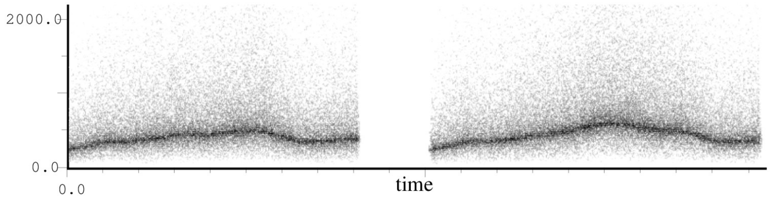

63 equal

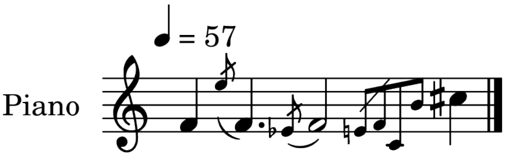

64 different

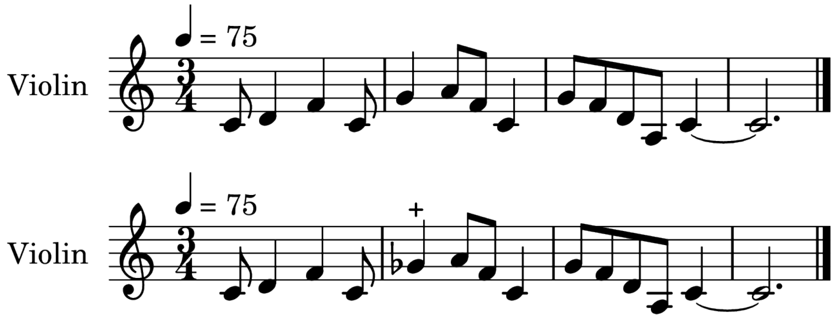

65 equal

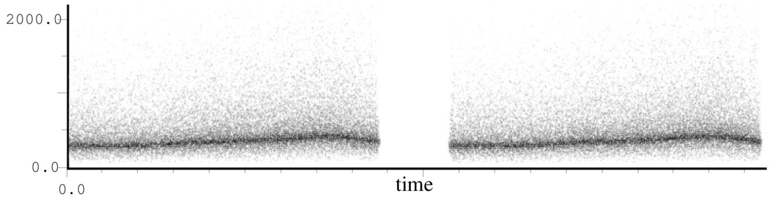

66 different

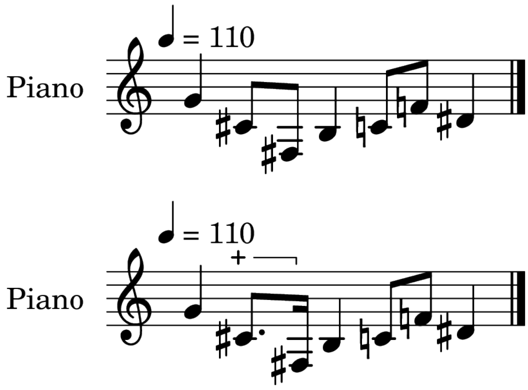

67 different

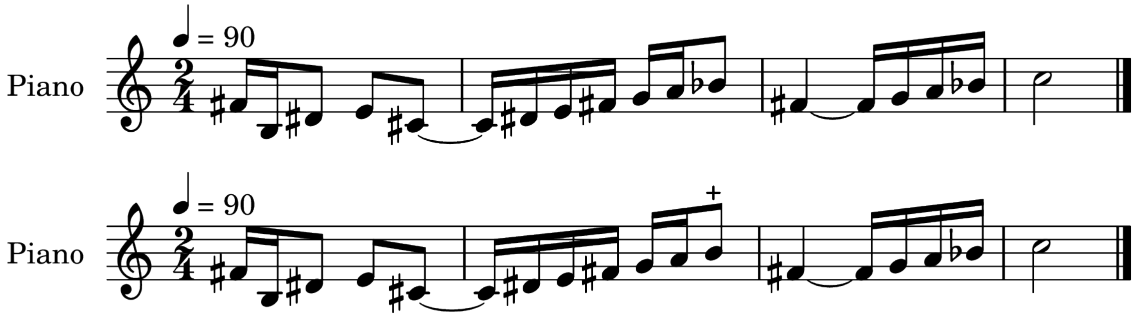

68 equal

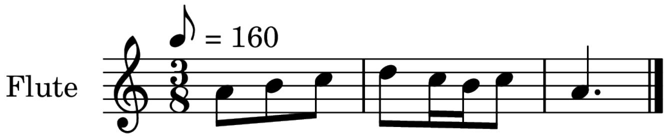

69 different

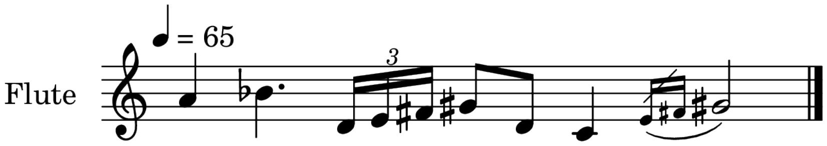

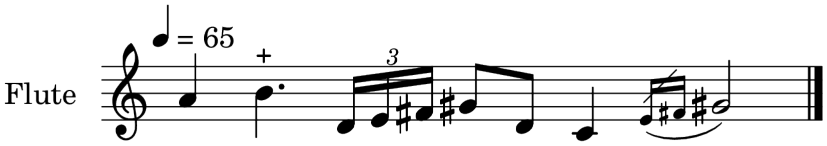

70 different

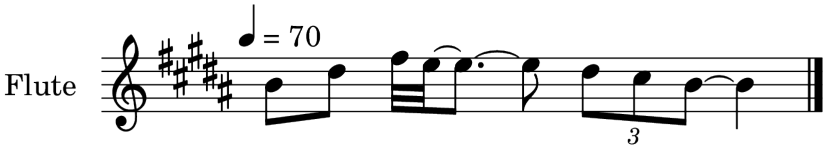

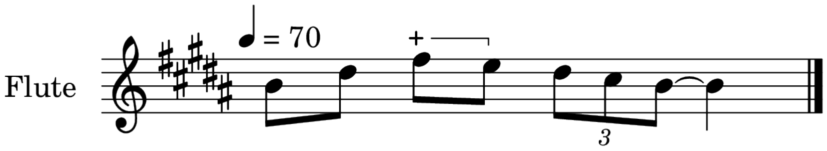

71 different

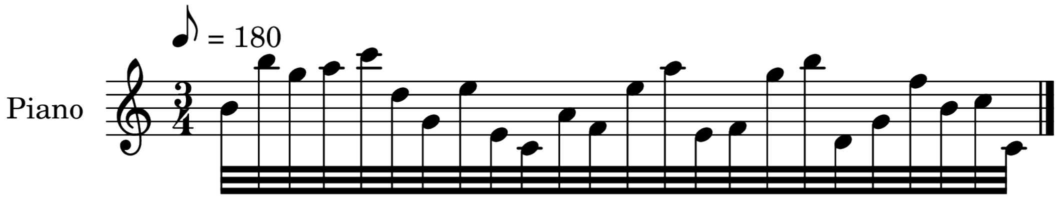

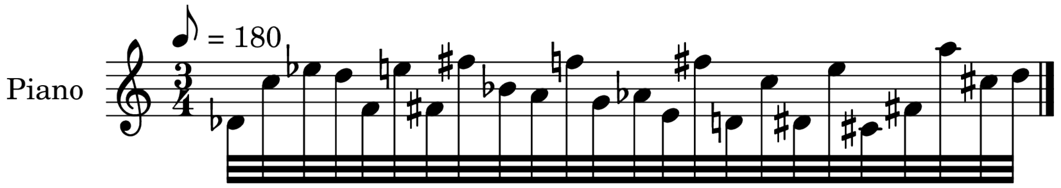

72 different

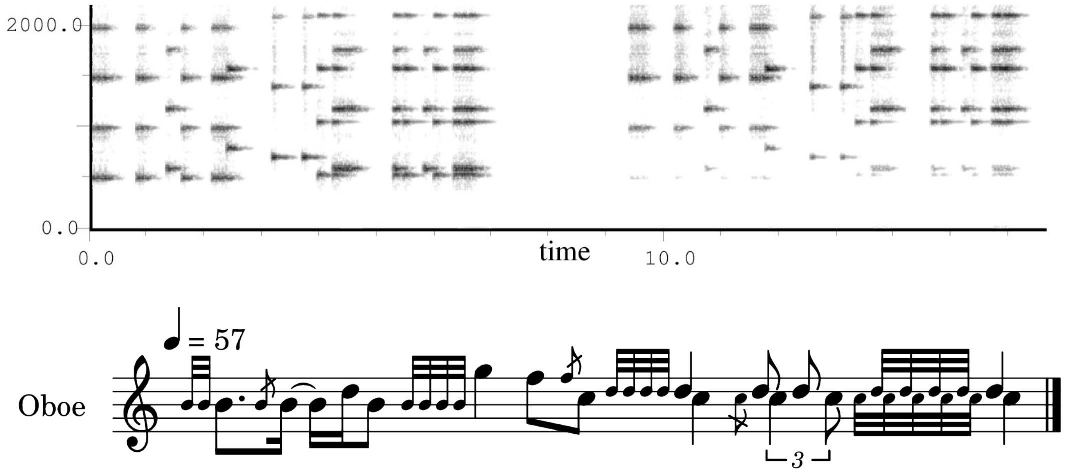

(2nd time with added filters)

73 equal

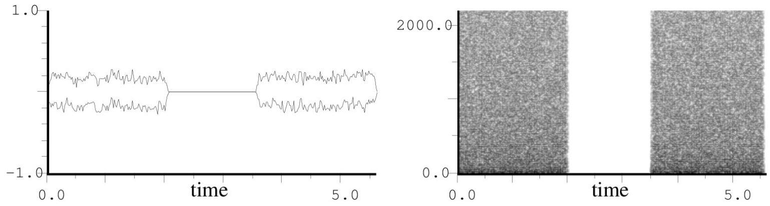

74 different

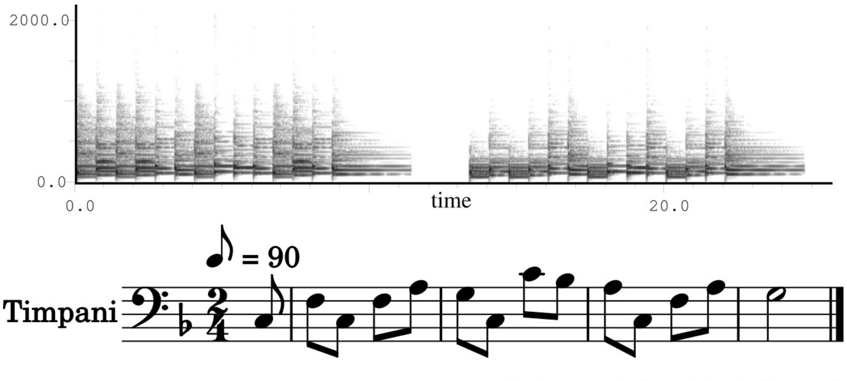

(2nd time with added filters)

75 different

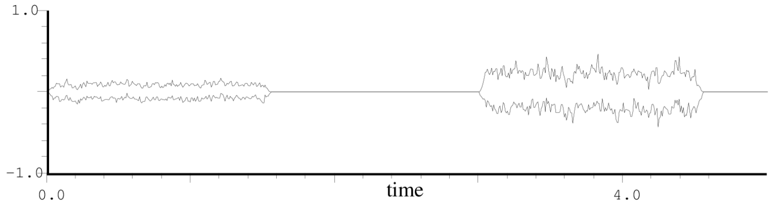

76 equal

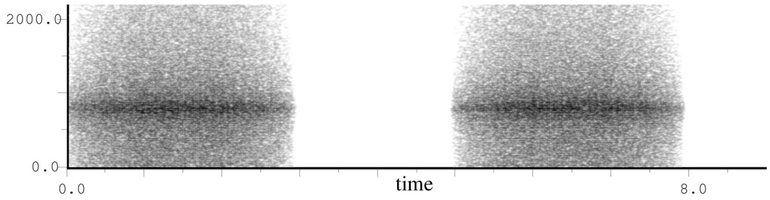

77 different

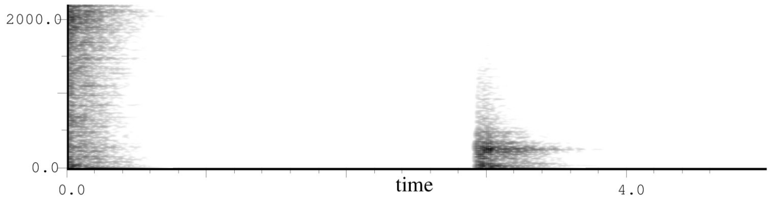

78 different

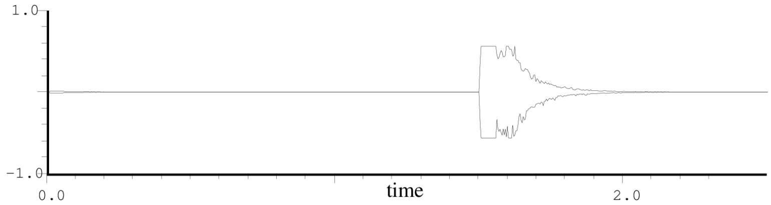

79 different

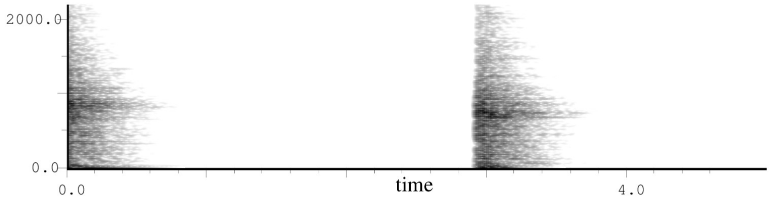

80 different

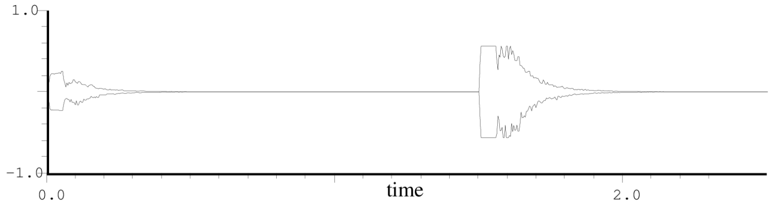

Supplement: Supplementary file 1 [file Image1.PDF]
